# Haploidentical Stem Cell Transplantation for Acute Myeloid Leukemia: Current Therapies, Challenges and Future Prospective

**DOI:** 10.3389/fonc.2021.758512

**Published:** 2021-10-28

**Authors:** Ying-Jun Chang, Xiang-Yu Zhao, Xiao-Jun Huang

**Affiliations:** ^1^ Peking University Institute of Hematology, Peking University People’s Hospital, Beijing, China; ^2^ National Clinical Research Center for Hematologic Disease, Beijing, China; ^3^ Beijing Key Laboratory of Hematopoietic Stem Cell Transplantation, Beijing, China; ^4^ Collaborative Innovation Center of Hematology, Peking University, Beijing, China

**Keywords:** acute myeloid leukemia, haploidentical stem cell transplantation, relapse, infection, graft-versus-leukemia-effect

## Abstract

Haploidentical stem cell transplantation (haplo-SCT), an alternative donor source, offers a curative therapy for patients with acute myeloid leukemia (AML) who are transplant candidates. Advances in transplantation techniques, such as donor selection, conditioning regimen modification, and graft-versus-host disease prophylaxis, have successfully improved the outcomes of AML patients receiving haplo-SCT and extended the haploidentical transplant indictions for AML. Presently, treating *de novo* AML, secondary AML, therapy-related AML and refractory and relapsed AML with haplo-SCT can achieve comparable outcomes to those of human leukocyte antigen (HLA)-matched sibling donor transplantation (MSDT), unrelated donor transplantation or umbilical cord blood transplantation. For some subgroups of AML subjects, such as patients with positive pretransplantation minimal/measurable residual disease, recent studies suggest that haplo-SCT might be superior to MSDT in decreasing relapse and improving survival. Unfortunately, for patients with AML after haplo-SCT, relapse and infections remain the causes of death that restrict further improvement in clinical outcomes. In this review, we discuss the recent advances and challenges in haplo-SCT for AML treatment, mainly focusing on unmanipulated haplo-SCT protocols. We provide an outlook on future prospects and suggest that relapse prophylaxis, intervention, and treatment, as well as infection prevention and therapy, are areas of active research in AML patients who receive haploidentical allografts.

## Introduction

**Figure 1:**
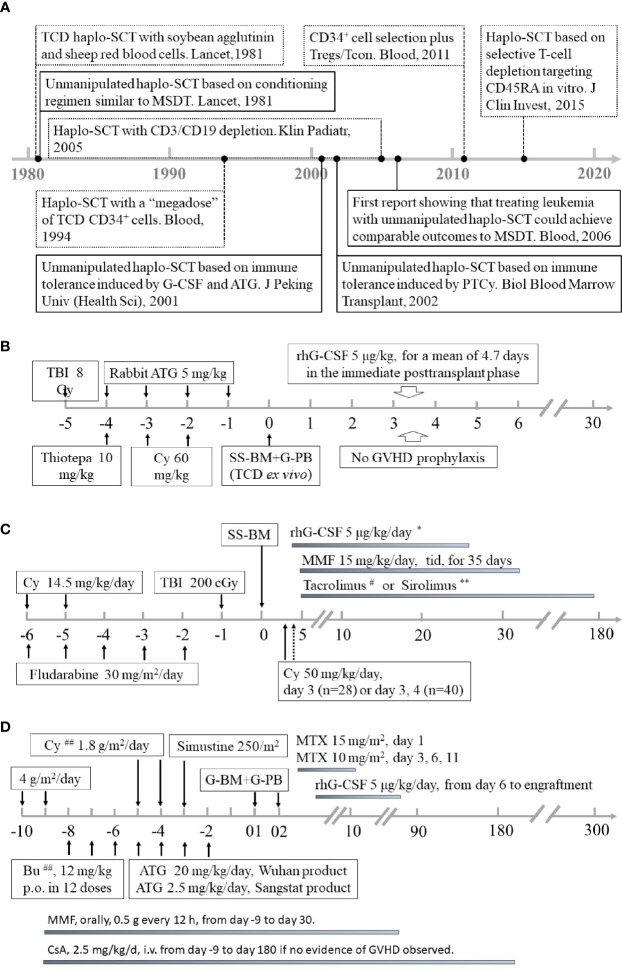
Summary of historical perspective and conditioning regimens of different haploidentical stem cell transplantation modalities for AML. **(A)** The historical perspective of haplo-SCT for AML. **(B)** University of Perugia: myeloablative conditioning and *ex vivo* TCD with “megadose” CD34^+^ cell allografts. **(C)** Peking University: myeloablative conditioning based on immune tolerance induced by G-CSF and ATG. **(D)** Johns Hopkins University: nonmyeloablative conditioning with high-dose PT/Cy. Panels **(B–D)** were adapted from Aversa et al. (Blood, 1994), Luznik et al. and Cieri et al. (Biol Blood Marrow Transplant,2008; Biol Blood Marrow Transplant,2015), and Huang et al. (Bone Marrow Transplant,2006), respectively. AML, acute myeloid leukemia; Haplo-SCT, haploidentical stem cell transplantation; Tregs, regulatory T cells; Tcon, conventional T cells; MSDT, human leukocyte antigen (HLA)-matched sibling donor transplantation; G-CSF, granulocyte colony-stimulating factor; ATG, anti-thymocyte globulin; PTCy, posttransplantation cyclophosphamide; TBI, Total body irradiation; SS-BM, steady-state bone marrow; G-PB, G-CSF mobilized peripheral stem cells; GVHD, graft-versus-host disease; MMF, mycophenolate mofetil; G-BM, G-CSF-stimulated bone marrow harvests; MTX, Methotrexate; Bu, Busulfan; CSA, Cyclosporin A ^*^Subcutaneous injection starting on Day 4 and continuing until recovery of neutrophils to >1000/μl for 3 days. ^#^Tacrolimus was initiated at a dose of 1 mg i.v. daily, adjusted to achieve a therapeutic level of 5–15 ng/mL, and then converted to oral form until discontinuation. If there was no active GVHD, tacrolimus was tapered off by Day 180. ^**^Sirolimus (orally, monitored 2 times each week to maintain a target therapeutic plasma level of 8 to 14 ng/mL during the first 2 months after transplantation, thereafter of 5 to 8 ng/mL until discontinuation). ^##^Patients 50 years old or older were conditioned with the same regimen as in **(D)**, except for lower dosages of Bu (6–8 mg/kg) and Cy (1.0 g/m^2^/d).

Allogeneic stem cell transplantation (allo-SCT) remains a curative therapy for patients with acute myeloid leukemia (AML) (1–19). However, the lack of human leukocyte antigen (HLA)-matched sibling donors (MSDs) restricts the wide use of allo-SCT in the clinic (20–22). To overcome the deficiency of donors, many efforts have been made to search for alternative donors (4, 6, 9, 23–27), including haploidentical donors (HIDs), HLA-matched unrelated donors (MUDs), and umbilical cord blood. Among these alternative donors, haploidentical allografts are the most attractive because successful application of haploidentical transplantation will ensure that almost every allograft candidate has a donor (4, 6, 9, 24–26). In the 1970s and 1980s, the clinical outcomes after bone marrow transplantation from HLA-mismatched family donors using a conditioning regimen similar to HLA-matched sibling donor transplantation (MSDT) in treating patients with AML were not acceptable because the long-term survival rate was less than 20% (28). In the 1990s, the introduction of a T-cell depletion (TCD) strategy followed by a myeloablative conditioning regimen improved the outcomes of haploidentical SCT (haplo-SCT) in the treatment of AML (29, 30). After 2000, the successful application of haplo-SCT based on immune tolerance induced by granulocyte colony-stimulating factor (G-CSF) and anti-thymocyte globulin (ATG, The Beijing Protocol) and haplo-SCT based on immune tolerance induced by posttransplant cyclophosphamide (PTCy, The Baltimore Protocol) in transplant candidates with hematological diseases (21, 31–36), such as patients with AML, allowed haploidentical allografts to be used worldwide, and it is a reality that almost everyone has a donor (**Figure 1**). The detailed history perspective and the biological differences of the abovementioned three haplo-SCT protocols has been extensively reviewed by others and by us elsewhere (22, 37–39).

In the past two decades, advances in the establishment of algorithms for best haploidentical donor selection (40–42), optimization of conditioning regimens (43–45), shifts from TCD grafts to unmanipulated bone marrow and/or peripheral blood harvests (3, 5, 21, 46, 47), enhancement of hematopoietic recovery through endothelial cell-directed N-acetyl-L-cysteine intervention and/or donor-specific antibody desensization (34, 48, 49), biomarker-directed graft-versus-host disease (GVHD) prophylaxis (50–52), minimal/measurable residual disease (MRD)-directed relapse intervention (7, 53), and approaches for enhancing immunologic recovery (54–56) have successfully improved the outcomes of patients with hematological malignancies, especially those with AML receiving haplo-SCT. Unfortunately, for subjects with AML who underwent haploidentical allografts, relapse and infections remain the causes of death that restrict further improvement in clinical outcomes (57, 58). In this review, we discussed the current therapies and challenges in haplo-SCT for AML treatment, mainly focusing on unmanipulated haploidentical transplant protocols. We provide an outlook on future prospects and suggest that relapse prophylaxis, intervention, and treatment, as well as infection prevention and therapy, are areas of active research in AML patients who receive haploidentical allografts.

## Current Therapies for AML With Haploidentical Allografts

### Extension of Haploidentical Transplant Indications for AML

The indications of AML for haploidentical protocols were significantly extended from adverse subjects to high-risk subgroup cases of favorable ones in the past two decades (5, 11, 13, 22, 59–61). First, for inter/high-risk *de novo* AML patients in complete remission 1 (CR1), our group demonstrated that for both adults and pediatric patients (5, 59, 60), haplo-SCT as postremission therapy achieved a lower CIR and superior survival in both MRD-negative and MRD-positive groups compared with those of patients treated with chemotherapy alone. Second, for favorable *de novo* AML cases in CR1, haplo-SCT could be used to improve outcomes in the following subgroup patients: i) t(8;21) AML cases who did not achieve major molecular remission (MMR)/MRD negativity, which were defined as >3-log reduction in RUNX1/RUNX1T1 transcripts (<0.4%) compared with the pretreatment baseline of 388% in Peking University Institute of Hematology, after the second consolidation therapy or those exhibiting the loss of MMR (defined as RUNX1/RUNX1T1 transcript levels >0.4% in MMR patients) within 6 months of achieving MMR (7, 61). ii) AML patients with NPM1 mutations (NPM1 m) failed to achieve a >4-log reduction in peripheral blood MRD after induction therapy (11). iii) For NPM1 wild-type standard-risk AML cases, those who were MRD-positive after second course induction (13). iv) CBFB-MYH11-positive AML patients with CBFB-MYH11/ABL levels >0.1% after two cycles of consolidation therapy (62). Third, other indications include secondary AML, therapy-related AML, and relapsed or refractory AML (R/R AML) (63, 66–75) (**Tables 1**–**3**).

**Table 1:** Recent studies comparing the outcomes of AML patients between haplo-SCT and other transplantation modalities.

Authors	Pros	Pt. N.	Age^*^	Remission status	SCT type	2–4 aGVHD	cGVHD	Relapse	NRM	LFS	OS	GRFS
Ciurea SO, et al. (4)	No	192	NA	CR2 (20%/35%)/Rel (34%/16%)	Haplo-SCT	16%/19%	30%/34% at 3 yr	44%/58% at 3 yr	14%/9% at 3 yr	NA	45%/46% at 3 yr	NA
		1982	NA	CR2 (20%/25%)/Rel (17%/22%)	MUDT	33%/28%	53%/52% at 3 yr	39%/42% at 3 yr	20%/23% at 3 yr	NA	50%/44% at 3 yr	NA
Wang Y, et al. (6)	Yes	231	28	Inter- or high-risk AML in CR1	Haplo-SCT	36%	42% at 1yr**	15% at 3 yr	13% at 3 yr	76% at 3 yr	79% at 3 yr	NA
		219	40	Inter- or high-risk AML in CR1	MSDT	13%	15% at 1yr	15% at 3 yr	8% at 3 yr	80% at 3 yr	82% at 3 yr	NA
Ruggeri A, et al. (64)	No	360	44	≧CR2 (28%)/AD (38%)^#^	Haplo-SCT	27%	29% at 2yr	—	—	32% at 2yr	—	NA
		558	45	≧CR2 (36%)/AD (19%)	UCBT	31%	24% at 2yr	HR=0.95	HR=1.16	38% at 2yr	HR=0.78	NA
Versluis J, et al. (65)	No	3511	50	Later CR (28%)	MSDT	22%	35% at 2yr	32% at 2yr^†^	15% at 2yr	53% at 2yr	59% at 2yr	NA
		1959	54	Later CR (22%)	MUDT (10/10)	26%	30% at 2yr	27% at 2yr	20% at 2yr	53% at 2yr	59% at 2yr	NA
		549	52	Later CR (26%)	MUDT (9/10)	28%	28% at 2yr	31% at 2yr	24% at 2yr	44% at 2yr	49% at 2yr	NA
		333	48	Later CR (24%)	UCBT	30%	19% at 2yr	30% at 2yr	29% at 2yr	41% at 2yr	44% at 2yr^††^	NA
		193	51	Later CR (25%)	Haplo-SCT	25%	29% at 2yr	22% at 2yr	26% at 2yr	52% at 2yr	57% at 2yr	NA
Santoro N, et al. (66)	No	250	65	≧CR2 (18%)/AD (44%)^#^	Haplo-SCT	31%	27% at 1yr	28% at 1yr	38% at 1yr	35% at 1yr	39% at 1yr	30% at 1yr
		2589	64.8	≧CR2 (17%)/AD (30%)	MUDT	33%	41% at 1yr	32% at 1yr	28% at 1yr	40% at 1yr	42% at 1yr	25% at 1yr
Baron F, et al. (62)	No	701	58	CR2 (14%)	MSDT	19%	50% at 2 yr	32% at 2 yr	13% at 2 yr	54% at 2 yr	59% at 2 yr	29% at 2 yr
		611	62	CR2 (20%)	MUDT	30%	51% at 2 yr	30% at 2 yr	20% at 2 yr	50% at 2 yr	56% at 2 yr	23% at 2 yr
		112	58	CR2 (34%)	Haplo-SCT	31%	30% at 2 yr	34% at 2 yr	22% at 2 yr	50% at 2 yr	53% at 2 yr	37% at 2 yr
		291	55	CR2 (39%)	UCBT	38%	28% at 2 yr	34% at 2 yr	16% at 2 yr	44% at 2 yr	43% at 2 yr	39% at 2 yr
Salvatore D, et al. (67)	No	185	50	Inter (66%)/High (34%)	Haplo-SCT	31%	33% at 2 yr	19% at 2 yr	23% at 2 yr^‡^	58% at 2 yr^‡^	68% at 2 yr^‡^	47% at 2 yr
		2469	50	Inter (76%)/High (24%)	MSDT	21%	35% at 2 yr	24% at 2 yr	10% at 2 yr	67% at 2 yr	76% at 2 yr	50% at 2 yr
Rashidi A, et al. (61)	No	336	NA	Secondary (27%)	Haplo-SCT	31%	26% at 3yr	38% at 3yr	19% at 3yr	43% at 3yr	48% at 3yr	NA
		869	NA	Secondary (24%)	MSDT	26%	56% at 3yr	38% at 3yr	14% at 3yr	48% at 3yr	55% at 3yr	NA
Ruggeri A, et al. (64)	No	163	56	CR1 (56%)	UCBT	33%	26% at 2 yr	33% at 2 yr	41% at 2 yr	26% at 2 yr	29% at 2 yr	17% at 2 yr^##^
		246	60	CR1 (44%)	Haplo-SCT	23%	26% at 2 yr	30% at 2 yr	34% at 2 yr	36% at 2 yr	41% at 2 yr	28% at 2 yr
Brissot E, et al. (68)	No	199	51.9	Refractory (41%)/relapse (59%)	Haplo-SCT	28.20%	19.30% at 2 yr	52% at 2 yr	25% at 2 yr	23% at 2 yr	29% at 2 yr	16% at 2 yr
		1111	52.4	Refractory (45%)/relapse (55%)	MUDT	30.60%	25.60% at 2 yr	46.30% at 2 yr	25.70% at 2 yr	28% at 2 yr	34.70% at 2 yr	16% at 2 yr
		383	51.7	Refractory (37%)/relapse (63%)	MMUDT	36.30%	27.40% at 2 yr	51.10% at 2 yr	26.70% at 2 yr	22.20% at 2 yr	27.60% at 2 yr	16% at 2 yr
Sanz J, et al. (69)	No	215	48	Inter (70%)/high (22%)	MSDT	17%	34% at 2 yr	33% at 2 yr	10% at 2 yr	57% at 2 yr	64% at 2 yr	45% at 2 yr
		235	47	Inter (60%)/high (34%)	MUDT	28%	32% at 2 yr	25% at 2 yr	14% at 2 yr	62% at 2 yr	68% at 2 yr	42% at 2 yr
		789	54	Inter (66%)/high (29%)	Haplo-SCT	26%	30% at 2 yr	23% at 2 yr	23% at 2 yr	54% at 2 yr	61% at 2 yr	46% at 2 yr
Battipaglia G, et al. (63)	No	389	52	Refractory (42%)/relapse (58%)	Haplo-SCT	28%	27% at 2 yr	50% at 2 yr	31% at 2 yr	19% at 2 yr	25% at 2 yr	18% at 2 yr
		1654	52	Refractory (56%)/relapse (44%)	MSDT	27%	42% at 2 yr	51% at 2 yr	22% at 2 yr	27% at 2 yr	32% at 2 yr	26% at 2 yr
Kharfan-Dabaja MA, et al. (70)	No	135	44	≧CR2(45%)/Rel(55%)	Haplo-SCT	27% at 180d	22% at 2 yr	48% at 2 yr	27% at 2 yr	29% at 2 yr	29% at 2 yr	19% at 2 yr
		320	46	≧CR2(50%)/Rel(50%)	MUDT	30% at 180d	32% at 2 yr	56% at 2 yr	26% at 2 yr	25% at 2 yr	31% at 2 yr	21% at 2 yr
Konuma T, et al. (22)	No	1102	51	CR1(76%)/≧CR2(24%)	UCBT	39%	31% at 3 yr	20% at 3 yr	25% at 3 yr	56% at 3 yr	59% at 3 yr	48% at 3 yr
		211	47	CR1(76%)/≧CR2(24%)	Haplo-SCT	30%	37% at 3 yr	22% at 3 yr	21% at 3 yr	58% at 3 yr	59% at 3 yr	43% at 3 yr

AML, acute myeloid leukemia; haplo-SCT, haploidentical stem cell transplantation; Pros, prospective; Pt., patient; N, number; SCT, stem cell transplantation; GVHD, graft-versus-host disease; aGVHD, acute GVHD; cGVHD, chronic GVHD; NRM, nonrelapse mortality; LFS, leukemia-free survival; OS, overall survival; GRFS, GVHD and relapse-free survival; NA, not available; Rel, relapse; CR2, complete remission 2; MUDT, human leukocyte antigen (HLA)-matched unrelated donor transplantation; yr, year; MSDT, HLA-matched sibling donor transplantation; AD, advanced disease; UCBT, umbilical cord blood transplantation; HR, hazard ratio; MMUDT, mismatched MUDT; d, day.

*the median age of patients.

**indicates P<0.001 compared with that of MSDT.

@indicates that patients with poor-risk AML were enrolled in this study.

^†^indicates that haplo-SCT and MUDT (10/10) had lower CIRs than MSDT (P<0.01 for all).

^††^indicates that UCBT experienced lower RFS than MUDT (10/10), haplo-SCT and MSDT (P<0.01 for all).

^#^indicates that the percentages of patients in the haplo-SCT group with ≥CR2 or AD were higher than those of the MUDT group (P<0.0001) or the UCB group (P<0.0001).

^‡^indicates P<0.01 compared with that of MSDT.

^##^indicates P=0.02 compared with that of haplo-SCT.

**Table 2:** Published PKIU studies comparing haplo-SCT with chemotherapy alone in adult and pediatric AML cases.

Author, Yr, Ref.	Pts (No.)	Diagnosis	Disease status	Treatment modality	Conditioning regimen	relapse	NRM	LFS	OS	Prospective study
**Huang XJ, et al. (** 5 **)**	58	Inter/high-risk	CR1	Haplo-SCT	MAC	12% at 4yr ^*^	0% at 4yr	74% at 4yr ^*^	78% at 4yr ^*^	Yes
	74	Inter/high-risk	CR1	Chemotherapy	NA	58% at 4yr	12% at 4yr	44% at 4yr	55% at 4yr	
**Zhu HH, et al. (** 7 **)**	40	High-risk	CR1	Haplo-SCT	MAC	21% at 5yr ^**^	NA	62% at 5yr ^**^	72% at 5yr ^**^	Yes
	29	High-risk	CR1	Chemotherapy	NA	79% at 5yr	NA	20% at 5yr	27% at 5yr	
**Lv M, et al. et al. (** 53 **)**	78	Inter-risk	CR1	Haplo-SCT	MAC	12% at 3 yr ^#^	15% at 3yr	73% at 3 yr ^#^	81% at 3 yr ^#^	Yes
	69	Inter-risk	CR1	Chemotherapy	NA	49% at 3 yr	3% at 3yr	47% at 3 yr	54% at 3 yr	
**Hu GH, et al. (** 55 **)**	27	High-risk	CR1	Haplo-SCT	MAC	18% at 5yr ^##^	HR=0.238	87% at 5yr ^##^	83% at 5yr	No
	28	High-risk	CR1	Chemotherapy	NA	50% at 5yr	P=0.032	62% at 5yr	71% at 5yr	
**Xue YJ, et al. (** 54 **)**	33	Inter-risk	CR1	Haplo-SCT	MAC	15% at 3yr ^†^	NA	82% at 3yr ^‡^	85% at 3yr	No
	47	Inter-risk	CR1	Chemotherapy	NA	33% at 3yr	NA	67% at 3yr	86% at 3yr	

PKIU, Peking University Institute of Hematology; MSDT, human leukocyte antigen-matched sibling donor transplantation; haplo-SCT, haploidentical stem cell transplantation; Yr, year; Ref., reference; No., number; aGVHD, acute graft-versus-host disease; NRM, nonrelapse mortality; LFS, leukemia-free survival; OS, overall survival; GRFS, GVHD and relapse-free survival; pre-MRD, pretransplantation minimal residual disease; Ad, advanced disease; MA, myeloablative; G-PB, granulocyte colony-stimulating factor (G-CSF)-mobilized peripheral blood harvests; G-BM, G-CSF simulated bone marrow harvests; NA, not available.

*P<0.01 for relapse, LFS and OS compared between haplo-SCT and chemotherapy.

**P<0.01 for relapse, LFS and OS compared between haplo-SCT and chemotherapy.

^#^P<0.001 for relapse, LFS and OS compared between haplo-SCT and chemotherapy.

^##^P<0.05 for relapse and LFS compared between haplo-SCT and chemotherapy.

^†^P=0.059.

^‡^indicate event-free survival.

**Table 3:** Studies on haploidentical allografts with superior graft-versus-leukemia effects to those of MSDT.

Author, Yr, Ref.	Pts (No.)	Dagnosis	Disease status	Transplant modality	Conditioning regimen	Stem cell source	2–4 aGVHD	Chronic GVHD	relapse	NRM	LFS	OS	GRFS
**Chang YJ, et al. (** 71 **)**	34	AML (pre-MRD+)	Inter+Ad (94%)	Haplo-SCT	MA (100%)	G-PB+G-BM (100%)	NA	NA	19% at 4 yr^*^	7% at 4 yr	74% at 4 yr^*^	83% at 4 yr^*^	NA
	107	AML (pre-MRD+)	Inter+Ad (82%)	MSDT	MA (100%)	G-PB+G-BM (100%)	NA	NA	55% at 4 yr	12% at 4 yr	33% at 4 yr	38% at 4 yr	NA
**Zhao XS, et al. (** 72 **)**	14	FLT3+ AML (pre-MRD+)	≧CR2 (29%)	Haplo-SCT	MA (100%)	G-PB+G-BM (100%)	36%	36% at 2 yr	31% at 2 yr^**^	8% at 2 yr	63% at 2 yr^**^	71% at 2 yr^**^	NA
	4	FLT3+ AML (pre-MRD+)	≧CR2 (0)	MSDT	MA (100%)	G-PB+G-BM (100%)	0	0% at 2 yr	75% at 1 yr	0% at 2 yr	33% at 2 yr	35% at 2 yr	NA
**Zhao XS, et al. (** 73 **)**	37	CBFB-MYH11+ AML (pre-MRD+)	≧CR2 (22%)	Haplo-SCT	MA (100%)	G-PB+G-BM (100%)	27%	72% at 2 yr	16% at 2 yr	14% at 2 yr	72% at 2 yr^#^	76% at 2 yr	NA
	9	CBFB-MYH11+ AML (pre-MRD+)	≧CR2 (22%)	MSDT	MA (100%)	G-PB+G-BM (100%)	11%	67% at 2 yr	41% at 2 yr	14% at 2 yr	51% at 2 yr	63% at 2 yr	NA
**Zheng FM, et al. (** 74 **)**	69	AML high-risk	≧CR2 (20.3%)	Haplo-SCT	MA (100%)	G-PB+G-BM (72.5%)	34.8%	35% at 3 yr	16% at 3 yr^##^	11% at 3 yr	73 at 3 yr	75 at 3 yr	NA
	23	AML high-risk	≧CR2 (26.1%)	MSDT	MA (100%)	G-PB+G-BM (60.9%)	13.0%	14% at 3yr	39% at 3 yr	0 at 3 yr	61% at 3 yr	73% at 3 yr	NA
**Guo HD, et al. (** 21 **)**	87	RUNX1/RUNX1T1+ AML (pre-MRD+)	≧CR2 (21%)	Haplo-SCT	MA (100%)	G-PB+G-BM (100%)	42%	62% at 5 yr	14% at 5 yr^†^	18% at 5 yr	68% at 5 yr^†^	70% at 5 yr^†^	NA
	48	RUNX1/RUNX1T1+ AML (pre-MRD+)	≧CR2 (4%)&	MSDT	MA (100%)	G-PB+G-BM (100%)	17%	64% at 5 yr	25% at 5 yr	27% at 5 yr	48% at 5 yr	50% at 5 yr	NA
**Yu S, et al. (** 75 **)**	83	High-risk AML	CR1 (100%)	Haplo-SCT	MA (100%)	G-PB+G-BM (100%)	40%	39% at 3 yr	14% at 3 yr	15% at 3 yr	71% at 3 yr	72% at 3 yr	63% at 3 yr^‡^
	106	High-risk AML	CR1 (100%)	MSDT	MA (100%)	G-PB (100%)	46%	51% at 3 yr	24% at 3 yr	10% at 3 yr	66% at 3 yr	68% at 3 yr	43% at 3 yr

MSDT, human leukocyte antigen-matched sibling donor transplantation; haplo-SCT, haploidentical stem cell transplantation; Yr, year; Ref., reference; No., number; aGVHD, acute graft-versus-host disease; NRM, nonrelapse mortality; LFS, leukemia-free survival; OS, overall survival; GRFS, GVHD and relapse-free survival; pre-MRD, pretransplantation minimal residual disease; Ad, advanced disease; MA, myeloablative; G-PB, granulocyte colony-stimulating factor (G-CSF)-mobilized peripheral blood harvests; G-BM, G-CSF simulated bone marrow harvests; NA, not available.

*P<0.01 for relapse, LFS and OS compared between haplo-SCT and MSDT.

**P<0.05 for relapse, LFS and OS compared between haplo-SCT and MSDT.

^#^P<0.05 for LFS compared between haplo-SCT and MSDT.

^##^P=0.027 for relapse compared between haplo-SCT and MSDT.

^†^P<0.05 for relapse, LFS and OS compared between haplo-SCT and MSDT.

^‡^P=0.035 for GRFS compared between haplo-SCT and MSDT.

The extension of indications promotes the use of haploidentical allografts in patients with AML worldwide (76, 77). According to the data of the Chinese Blood and Marrow Transplantation Register Group (CBMTRG) (76), haploidentical donors have been the first donor source for AML since 2013. The number of AML patients who received haplo-SCT reached nearly 2000 in 2015. In a recent survey by the European Society for Blood and Marrow Transplantation (EBMT) (77), the number of haplo-SCTs in Europe 2019 (n=1813) was listed as the third transplant modality for AML. In 2019, the number of haplo-SCTs was more than two thousand and comparable to that of MSDT according to the report of the Center for International and Marrow Transplant Research (CIBMTR), although fewer than 700 AML patients received haplo-SCT (78).

## Outcome Comparison Between Haplo-SCT and Other Transplant Modalities

In 2006, our group demonstrated for the first time that treating leukemia patients with haplo-SCT achieved comparable 2-year nonrelapse mortality (NRM), cumulative incidence of relapse (CIR), and 2-year probabilities of leukemia-free survival (LFS) and overall survival (OS) to those of MSDT (31), suggesting that haplo-SCT is a feasible approach with acceptable outcomes. Since then, a series of retrospective or prospective studies reported by others (4, 10, 26, 64–70, 74, 75, 79–87) and us (6, 88–90) compared the outcomes of AML patients who either received haplo-SCT or other allografts, including MSDT, HLA-matched unrelated donor transplantation (MUDT) and umbilical cord blood transplantation (UCBT). Here, we mainly focused on published studies that compared the outcomes between HIDs and other donors in the last five years (**Table 1**).

### Clinical Outcomes Between Haplo-SCT and MSDT

In 2015, researchers from China reported the results of a multicenter, prospective study that compared the outcomes of AML patients in CR1 who either underwent haplo-SCT (n=231) or MSDT (n=219) (6). Wang et al. (6) observed similar 3-year CIR (HR=1.06, *P*=0.85), NRM (HR=0.58, *P*=0.14), LFS (HR=0.83, *P*=0.42), and OS (HR=0.83, *P*=0.42) between the two transplant modalities. These results, together with other studies (69, 82, 91), suggest that haplo-SCT based on immune tolerance induced by G-CSF and ATG is a valid alternative as a postremission treatment of intermediate- or high-risk AML patients in CR1 lacking an identical donor. In 2019, after analyzing data obtained from the CIBMTR database, Rashidi et al. (79) demonstrated that patients with AML in CR1 who received PT-Cy-based haplo-SCT (n=336) had comparable outcomes in 3-year CIR (HR=0.88, *P*=0.27), NRM (HR=1.26, *P*=0.16), LFS (HR=1.06, *P*=0.50), and OS (HR=1.15, *P*=0.15) and significantly lower chronic GVHD (HR=0.38, *P*<0.01) than those who received MSDT (n=869). These results suggest that haplo-SCT is an alternative source for AML cases in CR1 (**Table 1**).

Except for the two studies reported by Rashidi et al. and Wang et al. (6, 79), other scholars also confirmed the similarity between haplo-SCT and MSDT in treating AML in CR2, secondary AML, poor-risk AML and refractory/relapsed AML (**Table 1**) (64, 70, 80). More recently, in a prospective multicenter cohort study, Yu et al. (92) showed that treating high-risk AML in CR1 with haploidentical allografts could significantly decrease the cumulative incidence of positive posttransplant MRD (18% *vs.* 42%, *P*<0.001) and increase the probability of 3-year GVHD and relapse-free survival (63% *vs.* 43%, *P*=0.035) compared with those who received allografts from MSDs. These results suggest that haplo-SCT has a stronger graft-versus-leukemia (GVL) effect than MSDT in high-risk AML patients in CR1 (92). Thus, increasing evidence supports the notion that haplo-SCT should be recommended as one of the optimal postremission therapy choices for transplant candidates with AML.

In a recent meta-analysis, Yang et al. (93) demonstrated that haplo-SCT, either the Baltimore protocol or the Beijing protocol, could achieve comparable 1-year CIR (OR, 0.83; *P*=0.180) and NRM (OR, 0.98; *P*=0.910) to those of MSDT in another meta-analysis, which included 24 studies and 11,359 cases. Overall, the literature published thus far (64, 70, 80) suggests that for patients with AML, MSDs remain the first choice when HIDs are also available due to the early delayed immune recovery and higher infection rate following haplo-SCT compared to those with MSDT (58, 94).

### Clinical Outcomes Between Haplo-SCT and MUDT

In 2009, Huang et al. (81) reported that treating hematological malignancies with haplo-SCT (n=219) could achieve comparable outcomes, including 2-year chronic GVHD (54% *vs.* 40%, *P*=0.17), CIR (12% *vs.* 18%, *P*=0.12), NRM (20% *vs.* 18%, *P*=0.98), LFS (67% *vs.* 61%, *P*=0.98) and OS (74% *vs.* 74%, *P*=0.74), to those of MUDT (n=78), although higher grades II to IV acute GVHD (HR=1.72, *P*=0.046) were observed in the haplo-SCT cohort. These preliminary data indicate that haplo-SCT could be an alternative source for patients with hematological malignancies who lack MSDs or MUDs (81). In another retrospective pair-matched comparative study of the EBMT database with data from the Beijing Protocol, Sun et al. (10) showed comparable outcomes between haplo-SCT and MUDT for treating AML patients in CR1, suggesting that HIDs could be an alternative stem cell source when a fully matched URD is not available.

For poor-risk AML in CR1, Versluis et al. (70) observed that haplo-SCT could achieve comparable outcomes to those of 10/10 matched MUDT but superior outcomes to those of 9/10 matched MUDT. Patients with refractory/relapsed AML who underwent haploidentical allografts experienced comparable outcomes to those of patients who received either MUDT or mismatched unrelated donor transplantation (MMUDT) (85). Ongoing prospective, randomized studies are performed to validate the disadvantages and advantages between haploidentical allografts and MUDT (NCT04067180 and NCT04232241), although current data (4, 24, 70, 83, 85, 86, 95) suggest that an HID is a valid option for high-risk AML patients in CR1 or with active disease as well as R/R AML.

Based on the results of the meta-analysis, Arcuri et al. (96) observed that treating hematological malignancies with PTCy-based haplo-SCT could achieve a similar OS rate (HR, 0.98) to MUDT. However, the incidence of all forms of GVHD (2–4 aGVHD, HR, 0.52; cGVHD, HR, 0.25) and NRM (HR, 0.85) was lower after haplo-SCT than after MUDT. Gagelmann et al. (97) showed that, compared with MMUDT, haplo-SCT with PTCy was associated with reduced all-cause mortality (OR, 0.75) and better outcomes (OR, 0.51). Overall, HIDs could be an alternative stem cell source for treating subjects with AML, especially poor-risk subjects, who lack MSDs in experienced centers due to easy access to first and second stem cell harvests, although the current algorithm suggests that MUDs (10/10) should be preferred to HIDs (42, 98).

### Clinical Outcomes Between Haplo-SCT and UCBT

In 2011, a 2 parallel multicenter phase 2 trial performed by Brunstein et al. (99) provided preliminary results indicating that the outcomes between double UCBT and haplomarrow transplantation with reduced intensity conditioning (RIC) regimens in treating leukemia and lymphoma are comparable to those reported after MUDT. However, from the point of view of graft acquisition and early direct charges, haplo-SCT may result in early cost savings over double UCBT and may be preferred by transplant centers and patients with more limited resources, as described by Kanate et al. (100)

In 2019, Ruggeri et al. (84) retrospectively analyzed the outcomes of 409 adults with secondary AML receiving either UCBT (n=163) or haplo-SCT (n=246) in EBMT centers. They observed a higher risk of grade II–IV acute GVHD (HR 1.9, *P*=0.009) and lower GHVD-free relapse-free survival (GRFS) (HR 1.57, *P*=0.007) after UCBT for subjects with AML compared to haploidentical allografts. These results indicate that haplo-SCT is associated with better GRFS and lower acute GVHD than UCBT in patients with secondary AML. For poor-risk AML patients, Versluis et al. (70) found that compared with UCBT, haplo-SCT was associated with higher RFS (52% *vs.* 41%, *P*<0.001).

More recently, in 2 parallel phase II trials, 368 patients aged 18 to 70 years with chemotherapy-sensitive lymphoma or acute leukemia in CR were randomly assigned to the UCBT group (n=186) or haplo-SCT group (n=182) (65). Prespecified analysis of secondary end points demonstrated lower 2-year NRM after haplo-SCT than that of UCBT (11% *vs.* 18%, *P*=0.04), which led to higher OS after haplo-SCT compared with that of UCBT (57% *vs.* 46%, *P*=0.04), but the PFS was comparable (35% *vs.* 41%, *P*=0.41). Fuchs et al. (65) suggested that although both donor sources extend access to RIC transplantation, analyses of secondary end points, including OS, favor HIDs.

In a recent meta-analysis, Wu et al. (71) found that haplo-SCT was associated with a lower NRM (0.72, 95% CI 0.64 to 0.80), leading to superior OS (OR, 0.74, 95% CI, 0.68 to 0.80) and PFS (0.77, 95% CI 0.72 to 0.83) compared with UCBT. Overall, the available published literature (65, 70, 84, 100), especially meta-analyses (71), suggests that haplo-SCT might be better than UCBT in treating AML.

**Figure 2:**
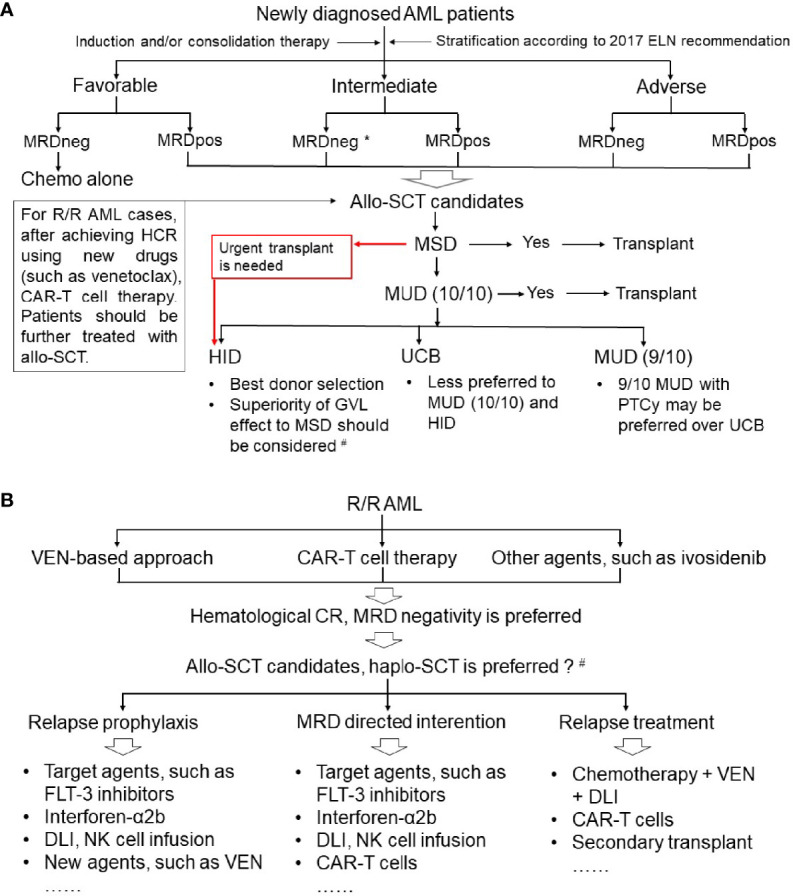
Position of haploidentical stem cell transplantation in the treatment of acute myeloid leukemia. **(A)** Newly diagnosed AML; **(B)** refractory or relapsed AML. AML, acute myeloid leukemia; ELN, European LeukemiaNet; MRD, minimal residual disease; MRDneg, negative MRD; MRDpos, positive MRD; Allo-SCT, allogeneic stem cell transplantation; R/R, refractory or relapsed; HCR, hematological complete remission; CAR-T, chimeric antigen receptor (CAR) T cell; MSD, human leukocyte antigen (HLA)-matched sibling donor; MUD, HLA-matched unrelated donor; HID, haploidentical donor; UCB, umbilical cord blood; PTCy, post-transplant cyclophosphamide; GVL, graft-versus-leukemia; VEN, venetoclax; Haplo-SCT, haploidentical SCT; DLI, donor lymphocyte infusion; NK cell, natural killer cell. *For intermediate-risk AML patients with MRD negative CR1, controversy remains regarding the selection of chemotherapy alone, autologous SCT or allo-SCT in patients. ^#^For pre-MRD positive AML cases, haplo-SCT had a stronger GVL effect compared with that of MSDT.

Overall, the landscape of allografts for hematological malignancies apparently changed with the position alteration of haplo-SCT in AML treatment (5, 11, 13, 22, 59–61). Most scholars agree that for patients with hematological malignancies, including AML, who lack MSDs and urgent transplantation, HIDs could be selected first. Impressively, based on the dataset of the Acute Leukemia Working Party of the EBMT registry, Dholaria et al. demonstrated that 9/10 MUDT with PTCy may be preferred over UCBT if a 10/10 matched unrelated donor is not available (72) (**Figure 2A**). Currently, a few retrospective studies compared the clinical outcomes between the Beijing Protocol and the Baltimore protocol in treating patients with hematological malignancies (73, 101, 102), however, the results among different studies remain controversial. Therefore, prospective, multicenter, randomized studies are needed.

## Challenges of Haploidentical Allograft in AML Treatment

According to the CIBMTR data, relapse remains an important challenge in AML patients who undergo haploidentical allografts. Infection is another challenge following haplo-SCT-based immune tolerance induced either by G-CSF and ATG or by PTCy (57, 58, 103, 104). Here, we discussed the recent emerging strategies for relapse or infection intervention or treatment for AML subjects.

### Could HIDs Be Selected First for Subgroup AML Patients to Decrease the Relapse Rate?

According to the current opinion, MSDs remain the first choice for transplant candidates with AML, although comparable outcomes were observed between haplo-SCT and MSDT (42, 105). However, we found that for all AML patients with positive pretransplantation MRD, haplo-SCT patients experienced a lower CIR and better LFS and OS than MDST patients (25, 106–109). These results suggest a stronger GVL effect of HIDs than MSDs. We further confirmed the stronger GVL effects after haplo-SCT than MSDT in AML subgroups with positive pretransplant MRD, including t(8;21) AML (25), Flt3 mutation-positive AML (110), and high-risk AML patients in CR1 (92) (**Table 3**). Interestingly, the stronger GVL effect of HIDs compared with MSDs was also confirmed in ALL patients with positive pre-MRD (111) and lymphoma subjects (105). More recently, a study from the Acute Leukemia Working Party of the EBMT showed that ALL patients treated with haplo-SCT experienced a significantly lower 2-year CIR than those of patients receiving MSDT (HR=0.63, *P*=0.002) (112), which provides new evidence supporting the stronger GVL effect of HIDs.

More recently, haploidentical and major histocompatibility (MHC)-matched transplant models were established by Guo et al. (25) after infusion of leukemia cells that carried the human AML-ETO or MLL-AF9 fusion gene to investigate the immune cell dynamic response during leukemia development *in vivo*. They showed that haplomatching the MHCs of leukemia cells with recipient mouse T cells prolonged leukemic mouse survival and reduced leukemia burden (25). The stronger GVL activity in the haplo-SCT group was mainly induced by decreased apoptosis and increased cytotoxic cytokine secretion, including tumor necrosis factor-α, interferon-γ, pore-forming proteins and CD107a secreted by T cells or natural killer (NK) cells (25).

Overall, in contrast to the traditional notion that MSDs remain the first choice, recent advances in haploidentical transplantation settings raise a new idea (92, 105, 107, 108, 110–114): for some subgroups of AML patients, HIDs might be chosen first (19), although further research is needed before this could be included in the donor selection algorithm (115, 116).

### Could the Best HIDs Be Selected to Decrease CIR of AML?

Donor characteristics are important variables for transplant outcome determination. In haplo-SCT settings, we and other researchers suggest a donor selection algorithm for which the key issue is that the younger the better (42, 117, 118). Regarding the best donor selection in AML patients treated with haplo-SCT, NK cell alloreactivity (KIR ligand mismatch between recipients and donors) was associated with better survival in AML patients who received haplo-SCT with ex vivo TCD. However, in unmanipulated haplo-SCT settings, KIR ligand mismatch was not associated with better survival of acute leukemia patients (42, 119, 120). Our group found that, compared to subjects with KIR ligand mismatch, cases with KIR ligand match were associated with rapid quantitative and functional NK cell recovery, which could contribute to lower CIR and better survival of AML cases treated with the Beijing Protocol (121). In a recent multicenter retrospective study, 1270 patients with acute lymphoma, including AML (n=1019) and ALL (n=251), received haplo-SCT using a myeloid ablative conditioning regimen or RIC. Cannani et al. (40) showed that for cases with age >40, donor age (>40) was correlated with higher NRM and inferior LFS and OS. Unfortunately, the current algorithm for the best HID selection is mainly based on the results obtained from the total patient population (41, 122). Therefore, multicenter, prospective studies are needed to answer the question of who is the best HID for AML patients, especially in unmanipulated haplo-SCT settings.

### Could We Incorporate Novel Methods With Haplo-SCT for R/R AML Treatment?

Venetoclax (VEN), a BCL-2 inhibitor, has been approved for unfit, older patients with AML (123, 124). In a recent study, sixty-eight patients, including newly diagnosed AML (ND AML) and R/R AML, were enrolled [phase IB (PIB), 16 (R/R); phase IIA (PIIA), 29 (ND); phase IIB (PIIB), 23 (R/R)]. Fludarabine (Flu), cytarabine (Ara-C), G-CSF, and idarubicin (IDA) + VEN (FLAG-IDA+VEN) were administered to all subjects. FLAG-IDA induction consisted of 28-day cycles of intravenous (iv) Flu (30 mg/m^2^) and Ara-c (1.5–2 g/m^2^ iv) on days (d) 2–6, IDA (iv; ND-AML: 8 mg/m^2^ d 4–6; R/R-AML: 6 mg/m^2^ d 4–5), and G-CSF (5 μg/kg d1–7). For patients in the PIB arm, VEN was administered as follows: 200 mg d1–21 (n=8), d1–14 (n=5); 400 mg d1–14 (n=3). For cases in the PIIA and PIIB arms, VEN was administered at 400 mg D1–14. After induction therapy, 67% of patients with R/R-AML (including 57% [n=8] subjects receiving prior allo-SCT) and 83% with sAML, t-AML, or ts-AML attained a CRc (CR+CRi). Eighteen (46%) R/R-AML cases, including 75% (n=6) of responding R/R patients who experienced a prior allo-SCT, were transitioned to allo-SCT. DiNardo et al. (125) further showed that for cases with R/R-AML, improved OS was observed in patients with consolidative allo-SCT in CRc versus without (median OS: NR [14 to not estimated (NE)] v 7 [4 to NE] months; *P*=0.009). This study provides a promising approach, combining FLAG-IDA+VEN with allografts, for the treatment of R/R AML.

To treat a patient with FUS-ERG^+^ AML who relapsed after allo-SCT within 3 months and resisted multiagent chemotherapy and donor lymphocyte infusion (DLI), Yao et al. (126) used donor-derived CD123-targeted CAR T cells (CART123) as part of a conditioning regimen for haplo-SCT. They observed a reduced blast level in BM within 2 weeks, which coincided with CAR copy expansion. After achieving full donor chimerism, this patient achieved CR with incomplete blood count recovery. These results suggest that CART123 in combination with haplo-SCT could be used as a therapy for relapsed AML subjects.

Overall, available data suggest that (125, 127–137), several other novel methods, such as ivosidenib, gilteritinib, flotetuzumab, quizartinib, CD33/CD3 bispecific T-cell engager antibody, and CAR- NK cells, could be successfully used for R/R AML patient therapy either alone or combined with allo-SCT, including haploidentical allografts (**Table 4**).

**Table 4:** Ongoing clinical trials focusing on patients with R/R AML or those who received haplo-SCT.

ClinicalTrials.gov Identifier	Randomized study	Estimated Study Completion Date	Aim of the study
**NCT04067180**	Yes	August 2028	To evaluate whether haplo-SCT is as good as URD SCT for the treatment of AML.
**NCT02782546**	No	January 30, 2024	To explore the whether CIML NK could improve LFS of AML patients receiving haplo-SCT.
**NCT04232241**	Yes	November 2024	To compare anti-leukemic activity between MUDT (10/10) and haplo-SCT for patients with AL.
**NCT03384225**	Yes	July 31, 2022	To evaluate if CBA could decrease relapse after haplo-SCT in HR/R AML compared with FBA.
**NCT03300492**	No	January 31, 2023	Safety, Feasibility of Pre-emptive therapy With *in Vitro* Expanded NK Cells in AML/MDS Patients receiving Haplo-HSCT (Phase I/II).
**NCT04678401**	No	October 31, 2023	Immunosuppression-free Treg-cell Graft-engineered haplo-SCT in R/R AML/MDS (Phase I).
**NCT04060277**	Yes	July 22, 2022	To Evaluate the Protective Function of CMV-MVA Triplex Vaccine in Adult Recipients of haplo-SCT.
**NCT04809181**	No	March 19, 2026	To investigate the efficacy of Aza plus VEN for Prevention of Relapse in MRD-Positive Post allo-SCT AML/MDS Patients.
**NCT04959903**	No	September 2026	To explore the Safety and the Efficacy of SMART101 Injection to Accelerate IR After TCD allo-SCT in Patients With AL (Phase I/II).
**NCT04599543**	No	November 15, 2023	To investigate the Safety and Efficacy of IL3 CAR-T Cell Therapy for R/R Acute Myeloid Leukemia.
**NCT04658004**	No	January 15, 2024	The Safety and Efficacy of NKG2D CAR-T Cell Therapy for Patients With R/R AML.
**NCT03473457**	No	December 31, 2022	The CAR-T cells (single CAR-T or double CAR-T cells with CD33,CD38,CD56,CD123,CD117,CD133,CD34 or Mucl) for R/R AML
**NCT04014881**	No	July 1, 2022	To evaluate the safety and efficacy of anti-CD123 CAR-T cells in patients with R/R CD123^+^ AML.
**NCT03971799**	No	December 2024	To determine The safety and feasibility of anti-CD33 CAR-T cells in children and AYAs with R/R AML.
**NCT04010877**	No	December 31, 2023	The feasibility, safety and efficacy of multiple CAR T-cell therapy targeting CD123 or CD33 in patients with R/R AML.
**NCT04272125**	No	July 1, 2023	To evaluate the efficacy and safety of CD123-targeted CAR-T cell therapy for patients with R/R AML.
**NCT04835519**	No	April 8, 2024	To evaluate safety and tolerability of functionally enhanced CD33 CAR-T cells in patients with R/R AML.
**NCT04219163**	No	July 31, 2038	CAR T-cells for The Treatment of AML Expressing CLL-1 Antigen.
**NCT04678336**	No	January 2036	To explore the safety, feasibility, and efficacy of CART123 cells in pediatric subjects with R/R AML (Phase 1).
**NCT04762485**	No	February 28, 2024	This is a phase 1/2 study to evaluate the efficacy and safety of CAR-T cells targeting CD7 for patients with R/R CD7 positive AL.
**NCT04766840**	No	December 1, 2023	To Evaluate the Safety and Efficacy of Donor-derived CAR-T Cells for patients with R/R AML.
**NCT03631576**	No	August 10, 2021	This study aims to assess the safety and toxicity of CD123/CLL1 CAR-T Cells to patients with R/R AML.
**NCT04318678**	No	July 1, 2025	To explore the safety of autologous, CD123-CAR T cells in patients (≤21 years) with R/R CD123+ AML.
**NCT04803929**	No	March 1, 2026	To investigate the safety and efficacy of novel ILT3-targeted CAR-T cell therapy for patients with R/R AML (M4/M5).
**NCT04789408**	No	January 2024	A Phase 1 Open-label, Multicenter Study Evaluating an Autologous Anti-CLL-1 CAR T-cell therapy in Subjects With R/R AML.
**NCT03190278**	No	October 2022	Phase I first-in-human study evaluating the safety and efficacy of UCART targeting CD123 in patients with R/R AML.

AML, acute myeloid leukemia; Haplo-SCT, haploidentical stem cell transplantation; URD SCT, unrelated donor SCT; CIML NK, cytokine-induced memory-like natural killer cells; LFS, leukemia-free survival; AL, acute leukemia; MUDT, human leukocyte antigen-matched unrelated donor transplantation; CBA, cladribine-based conditioning; FBA, fludarabine-based conditioning regimen; HR, high-risk; R, refractory; R/R, relapsed/refractory; Treg, regulatory T cells; CAR-T, chimeric antigen receptor (CAR)-expressing T cells; Aza, azacitidine; VEN, venetoclax; MRD, minimal residual disease; TCD, T cell depleted; SMART101, human T lymphocyte progenitor; IR, immune recovery; IL-3, interleukin-3.

### Could We Incorporate Other Approaches for AML Relapse Treatment and Prevention After haplo-SCT?

The outcomes of AML patients who relapse after allografts remain poor, with a 5-year OS of less than 20%, and either DLI or second allo-SCT is prescribed (138). More recently, Cui et al. (139) enrolled 6 AML patients who relapsed after transplantation. The median percentage of CD38 expression on blasts in the bone marrow of these patients was 95% before CD38-targeted CAR-T cell (CAR-T-38, 4 from autologous and 2 from donors) treatment. Four of six (66.7%) patients achieved CR or CR with incomplete count recovery (CRi) 4 weeks after the initial CAR-T-38 cell therapy. The CIR at 6 months was 50%. The median times of OS and LFS of these cases were 7.9 and 6.4 months, respectively. One patient who relapsed 117 days after the first CAR-T-38 treatment achieved remission after the second CAR-T-38 cell infusion. The side effects of these patients were manageable. There were no off-target effects on monocytes and lymphocytes. Although a limited number of cases and a relatively short follow-up time were presented by Cui et al. (139), their preliminary data highlight the clinical utility and safety of CAR-T-38 cell therapy in treating relapsed AML following allo-SCT. Several trials (NCT02782546, 04024761, 03300492) investigating the feasibility of immunotherapy with NK cells are ongoing (**Table 4**).

Considering the poor outcomes of HR-AML, a number of strategies for relapse prophylaxis or prevention have been used in the clinic (140). In a phase II, open-label, multicenter, randomized controlled trial (141), 204 HR-AML subjects with negative MRD who had received allo-SCT (mainly haplo-SCT, n=148) 60–100 days before were randomly (1:1) assigned to either no intervention (non–G-Dec group) or rhG-CSF combined with minimal dose Dec (G-Dec group: 100 mg/m^2^ of rhG-CSF on Days 0–5 and 5 mg/m^2^ of Dec on Days 1–5). Gao et al. (141) observed that patients in the G-Dec group experienced a lower 2-year CIR (15.0% *vs.* 38.3%, *P*=0.01) accompanied by rapid recovery of CD8^+^ T cells, NK cells, and regulatory T cells compared with patients in the non–G-Dec group, both of which led to higher LFS (HR=0.38, *P*<0.01) and OS (HR=0.45, *P*=0.01). No differences in the 2-year chronic GVHD without relapse between the two groups (23.0% *vs.* 21.7%, *P*=0.81) were shown. The authors (141). suggest that rhG-CSF combined with minimal-dose Dec maintenance therapy following transplantation can reduce the CIR, leading to the acquisition of GVL effects and immune tolerance.

Impressively, data from two independent randomized trials (15, 142) show that sorafenib maintenance posttransplantation, including haplo-SCT, prevents disease relapse in patients with FLT3-ITD AML both with negative or positive MRD after allograft transplantation, resulting in an OS benefit. A previous study by Mathew et al. (143), in allograft settings, showed that sorafenib increased IL-15 production by FLT3-ITD^+^ leukemia cells. IL-15 further caused an increase in CD8^+^CD107a^+^IFN-γ^+^ T cells with high levels of Bcl-2 and reduced PD-1 levels, and this cell subset could eradicate leukemia in secondary recipients. These studies (15, 142) provided strong evidence indicating that targeted posttransplant maintenance therapy should be a new treatment paradigm for AML, although questions remain. Moreover, additional studies are needed to investigate the optimal initial time and duration of sorafenib maintenance after allo-SCT as well as to elucidate the underlying mechanisms of sorafenib activity in the allograft setting.

In summary, the successful application of new strategies following allografting (15, 137, 142, 144), such as rhG-CSF combined with minimal-dose Dec, Aza plus VEN (NCT04809181), and targeted agent maintenance, could help AML patients avoid hematological relapse as much as possible, thus decreasing the CIR and improving the survival rate (**Figure 2B**).

### Could Infections Be Effectively Prevented Using Adoptive Cell Therapy?

In haplo-SCT with the G-CSF modality, the cumulative incidence of cytomegalovirus (CMV) DNAemia varies from 63.7 to 66.1% and remains one of the main causes of morbidity and mortality. BKV and EBV infection are also frequent in haplo-SCT and a risk factor for worse survival except for CMV infection (58, 145). For cases with refractory CMV infection who failed to respond to ganciclovir, foscarnet, and cidofovir, adoptive T-cell therapies, such as CMV-specific T-cells (CMV CTLs), represent a promising approach (146, 147). Using a humanized HCMV-infected mouse model, our group further elucidated that systemic HCMV infection could be combated after first-line therapy with CMV CTLs *via in vivo* promotion of the recovery of graft-derived endogenous CMV CTLs (55). These studies provide substantial evidence suggesting that refractory CMV infection could be successfully treated by adoptive transfer of CMV CTLs. Future studies should focus on risk factor-directed intervention or the development of new drugs for CMV infections in haplo-SCT settings.

Olson et al. (148) performed a clinical trial in which HLA-matched third-party BKV-specific CTLs were infused into 59 patients who developed BKV-HC following allo-SCT. They observed a rapid response to BKV-CTL infusion. The Day 14 and Day 45 overall response rates were 67.7% and 81.6%, respectively. No patient lost a previously achieved response. There were no cases of *de novo* grade III or IV GVHD, graft failure, or infusion-related toxicities. BKV-CTLs were observed in patient blood samples up to 3 months postinfusion, and their *in vivo* expansion predicted a clinical response. This study suggests that off-the-shelf BKV-CTLs are a safe and effective therapy for the management of patients with BKV-HC after allo-SCT (148). Therefore, rapid reconstitution of immunity to a broad range of viral and fungal infections can be achieved using a multipathogen-specific T-cell product (147, 149).

In summary, recent studies (147) showed some promising preliminary data and ongoing clinical trials on AML relapse prophylaxis, intervention, and treatment (**Figure 2B** and **Table 4**), as well as infection prevention and therapy. Both of these factors will pave the way for outcome improvements for patients with AML who undergo haploidentical allografts.

## Future Prospects

In the next five to ten years, the issue of relapse and infections remains to be solved, although haplo-SCT has been rapidly expanded in AML treatment (5, 11, 13, 22, 59–61). Regarding relapse, elucidating the mechanisms underlying leukemia recurrence remains the most important way to find novel targets for intervention or treatment of relapse. Recently, the application of single-cell sequencing techniques has been used for the following purposes (150–160): i) identifying differentiated AML cells with immunosuppressive properties; ii) dissecting the clonal heterogeneity of AML; iii) providing novel insights into the clonal evolution and resistance mechanisms of leukemia cells; iv) identifying novel targets for AML therapy; and v) highlighting the profound impact of AML on NK cell heterogeneity. These advances provide new clues and suggest that we could further discover new mechanisms underlying leukemia relapse after transplantation based on the abovementioned new techniques as well as *in vitro* and *in vivo* functional experiments.

In addition, based on current available data (25, 106, 108, 113, 140, 161), at different timepoints (pre- and posttransplantation), realization of individual therapy of AML by combining haplo-SCT with other novel methods (113), such as CAR-T therapy, target agents, and others, should be investigated. Could changing positive MRD to negative MRD pretransplantation further improve clinical outcomes? Which is the best method for positive pretransplant MRD eradication? For patients with intermediate- or adverse-stratification, should maintenance after transplant be given routinely? To answer these questions, prospective, multicenter, randomized clinical trials are urgently needed.

Infections, especially viral infections, are of concern. Both in the Beijing Protocol and the Baltimore Protocol, the delayed reconstitution of CMV-specific CTLs and NK cells was associated with CMV reactivation (58, 145). Therefore, enhancing CMV-specific CTL and NK cell recovery represents a future direction for virus infection prevention, including CMV, EBV, and BKV. Unfortunately, overcoming the functional impairments of adaptive NK cells to produce IFN-γ (162), a phenomenon due to the virus-induced expression of lymphocyte activation gene 3 and programmed cell death protein 1 checkpoint inhibitors, remains to be investigated. In addition, a phase II multicenter, randomized trial is ongoing to investigate the protective function of the CMV-MVA triplex vaccine in adult recipients who received haplo-SCT (NCT 04161885).

## Conclusion

Recent advances in haploidentical allografts have significantly changed their position in AML treatment (5, 11, 13, 22, 59–61). Their combination with novel therapies, such as CAR-T cells and Ven, could make more R/R patients with AML eligible for curative haplo-SCT who previously experienced poor outcomes when receiving allografts in relapse or NR status (125). Ongoing studies focusing on relapse, infections, and hematopoietic and immunological reconstitution enhancement would further improve haploidentical transplant outcomes of AML. In the long term, biomarkers (163, 164), such as MRD, directed donor selection (108), conditioning selection (14), and immunological enhancement for relapse intervention (165), will help us realize precision medicine in the setting of haplo-SCT for treating patients with AML.

## Author Contributions

Y-JC and X-JH designed the study. All authors contributed to data interpretation, manuscript preparation, and approval of the final version.

## Funding

This work was partly supported by grants from the Beijing Municipal Science and Technology Commission (Z181100009618032) and the National Key Research and Development Program of China (2017YFA0104500).

## Conflict of Interest

The authors declare that the research was conducted in the absence of any commercial or financial relationships that could be construed as a potential conflict of interest.

## Publisher’s Note

All claims expressed in this article are solely those of the authors and do not necessarily represent those of their affiliated organizations, or those of the publisher, the editors and the reviewers. Any product that may be evaluated in this article, or claim that may be made by its manufacturer, is not guaranteed or endorsed by the publisher.

## References

[1] Dohner H Estey E Grimwade D Amadori S Appelbaum FR Büchner T. Diagnosis and Management of AML in Adults: 2017 ELN Recommendations From an International Expert Panel. Blood (2017) 129:424–47. doi: 10.1182/blood-2016-08-733196 PMC5291965 27895058

[2] Short NJ Rytting ME Cortes JE. Acute Myeloid Leukaemia. Lancet (2018) 392:593–606. doi: 10.1016/S0140-6736(18)31041-9 30078459 PMC10230947

[3] Huang XJ Liu DH Liu KY Xu LP Chen H Han W. Treatment of Acute Leukemia With Unmanipulated HLA-Mismatched/Haploidentical Blood and Bone Marrow Transplantation. Biol Blood Marrow Transplant (2009) 15:257–65. doi: 10.1016/j.bbmt.2008.11.025 19167686

[4] Ciurea SO Zhang MJ Bacigalupo AA Bashey A Appelbaum FR Aljitawi OS. Haploidentical Transplant With Posttransplant Cyclophosphamide vs Matched Unrelated Donor Transplant for Acute Myeloid Leukemia. Blood (2015) 126:1033–40. doi: 10.1182/blood-2015-04-639831 PMC4543223 26130705

[5] Huang XJ Zhu HH Chang YJ Xu LP Liu DH Zhang XH. The Superiority of Haploidentical Related Stem Cell Transplantation Over Chemotherapy Alone as Postremission Treatment for Patients With Intermediate- or High-Risk Acute Myeloid Leukemia in First Complete Remission. Blood (2012) 119:5584–90. doi: 10.1182/blood-2011-11-389809 22535659

[6] Wang Y Liu QF Xu LP Liu KY Zhang XH Ma X. Haploidentical vs Identical-Sibling Transplant for AML in Remission: A Multicenter, Prospective Study. Blood (2015) 125:3956–62. doi: 10.1182/blood-2015-02-627786 25940714

[7] Zhu HH Zhang XH Qin YZ Liu DH Jiang H Chen H. MRD-Directed Risk Stratification Treatment May Improve Outcomes of T(8;21) AML in the First Complete Remission: Results From the AML05 Multicenter Trial. Blood (2013) 121:4056–62. doi: 10.1182/blood-2012-11-468348 23535063

[8] Mariotti J Raiola AM Evangelista A Carella AM Martino M Patriarca F. Impact of Donor Age and Kinship on Clinical Outcomes After T-Cell-Replete Haploidentical Transplantation With PT-Cy. Blood Adv (2020) 4:3900–12. doi: 10.1182/bloodadvances.2020001620 PMC7448598 32813875

[9] Lee CJ Savani BN Mohty M Labopin M Ruggeri A Schmid C. Haploidentical Hematopoietic Cell Transplantation for Adult Acute Myeloid Leukemia: A Position Statement From the Acute Leukemia Working Party of the European Society for Blood and Marrow Transplantation. Haematologica (2017) 102:1810–22. doi: 10.3324/haematol.2017.176107 PMC5664385 28883081

[10] Sun Y Beohou E Labopin M Volin L Milpied N Yakoub-Agha I. Unmanipulated Haploidentical Versus Matched Unrelated Donor Allogeneic Stem Cell Transplantation in Adult Patients With Acute Myelogenous Leukemia in First Remission: A Retrospective Pair-Matched Comparative Study of the Beijing Approach With the EBMT Database. Haematologica (2016) 101:e352–4. doi: 10.3324/haematol.2015.140509 PMC4967588 27081180

[11] Balsat M Renneville A Thomas X de Botton S Caillot D Marceau A. Postinduction Minimal Residual Disease Predicts Outcome and Benefit From Allogeneic Stem Cell Transplantation in Acute Myeloid Leukemia With NPM1 Mutation: A Study by the Acute Leukemia French Association Group. J Clin Oncol (2017) 35:185–93. doi: 10.1200/JCO.2016.67.1875 28056203

[12] Craddock C Slade D De Santo C Wheat R Ferguson P Hodgkinson A. Combination Lenalidomide and Azacitidine: A Novel Salvage Therapy in Patients Who Relapse After Allogeneic Stem-Cell Transplantation for Acute Myeloid Leukemia. J Clin Oncol (2019) 37:580–8. doi: 10.1200/JCO.18.00889 PMC6494237 30653424

[13] Freeman SD Hills RK Virgo P Khan N Couzens S Dillon R. Measurable Residual Disease at Induction Redefines Partial Response in Acute Myeloid Leukemia and Stratifies Outcomes in Patients at Standard Risk Without NPM1 Mutations. J Clin Oncol (2018) 36:1486–97. doi: 10.1200/JCO.2017.76.3425 PMC5959196 29601212

[14] Hourigan CS Dillon LW Gui G Logan BR Fei M Ghannam J. Impact of Conditioning Intensity of Allogeneic Transplantation for Acute Myeloid Leukemia With Genomic Evidence of Residual Disease. J Clin Oncol (2020) 38:1273–83. doi: 10.1200/JCO.19.03011 PMC7164487 31860405

[15] Xuan L Wang Y Huang F Fan Z Xu Y Sun J. Sorafenib Maintenance in Patients With FLT3-ITD Acute Myeloid Leukaemia Undergoing Allogeneic Haematopoietic Stem-Cell Transplantation: An Open-Label, Multicentre, Randomised Phase 3 Trial. Lancet Oncol (2020) 21:1201–12. doi: 10.1016/S1470-2045(20)30455-1 32791048

[16] Baron F Efficace F Cannella L Muus P Trisolini S Halkes CJM. Impact of the Type of Anthracycline and of Stem Cell Transplantation in Younger Patients With Acute Myeloid Leukaemia: Long-Term Follow Up of a Phase III Study. Am J Hematol (2020) 95:749–58. doi: 10.1002/ajh.25795 32233095

[17] Baron F Efficace F Cannella L Willemze R Vignetti M Muus P. Long-Term Follow-Up of a Trial Comparing Post-Remission Treatment With Autologous or Allogeneic Bone Marrow Transplantation or Intensive Chemotherapy in Younger Acute Myeloid Leukemia Patients. Haematologica (2020) 105:e13–6. doi: 10.3324/haematol.2019.221333 PMC6939505 31097634

[18] Cornelissen JJ van Putten WL Verdonck LF Theobald M Jacky E Daenen SM. Results of a HOVON/SAKK Donor Versus No-Donor Analysis of Myeloablative HLA-Identical Sibling Stem Cell Transplantation in First Remission Acute Myeloid Leukemia in Young and Middle-Aged Adults: Benefits for Whom? Blood (2007) 109:3658–66. doi: 10.1182/blood-2006-06-025627 17213292

[19] Zhang XH Chen J Han MZ Huang H Jiang EL Jiang M. The Consensus From The Chinese Society of Hematology on Indications, Conditioning Regimens and Donor Selection for Allogeneic Hematopoietic Stem Cell Transplantation: 2021 Update. J Hematol Oncol (2021) 14:145. doi: 10.1186/s13045-021-01159-2 34526099 PMC8441240

[20] Aversa F Terenzi A Tabilio A Falzetti F Carotti A Ballanti S. Full Haplotype-Mismatched Hematopoietic Stem-Cell Transplantation: A Phase II Study in Patients With Acute Leukemia at High Risk of Relapse. J Clin Oncol (2005) 23:3447–54. doi: 10.1200/JCO.2005.09.117 15753458

[21] Huang XJ Liu DH Liu KY Xu LP Chen H Han W. Haploidentical Hematopoietic Stem Cell Transplantation Without *In Vitro* T-Cell Depletion for the Treatment of Hematological Malignancies. Bone Marrow Transplant (2006) 38:291–7. doi: 10.1038/sj.bmt.1705445 16883312

[22] Kanakry CG Fuchs EJ Luznik L. Modern Approaches to HLA-Haploidentical Blood or Marrow Transplantation. Nat Rev Clin Oncol (2016) 13:10–24. doi: 10.1038/nrclinonc.2015.128 26305035 PMC4695979

[23] Barker JN Davies SM DeFor T Ramsay NK Weisdorf DJ Wagner JE. Survival After Transplantation of Unrelated Donor Umbilical Cord Blood is Comparable to That of Human Leukocyte Antigen-Matched Unrelated Donor Bone Marrow: Results of a Matched-Pair Analysis. Blood (2001) 97:2957–61. doi: 10.1182/blood.V97.10.2957 11342417

[24] Cho BS Min GJ Park S Park SS Shin SH Yahng SA. Haploidentical vs Matched Unrelated Donor Transplantation for Acute Myeloid Leukemia in Remission: A Prospective Comparative Study. Am J Hematol (2021) 96:98–109. doi: 10.1002/ajh.25993 32905642

[25] Guo H Chang YJ Hong Y Xu LP Wang Y Zhang XH. Dynamic Immune Profiling Identifies the Stronger Graft-Versus-Leukemia (GVL) Effects With Haploidentical Allografts Compared to HLA-Matched Stem Cell Transplantation. Cell Mol Immunol (2021) 18:1172–85. doi: 10.1038/s41423-020-00597-1 PMC8093297 33408344

[26] Konuma T Kanda J Yamasaki S Harada K Shimomura Y Terakura S. Single Cord Blood Transplantation Versus Unmanipulated Haploidentical Transplantation for Adults With Acute Myeloid Leukemia in Complete Remission. Transplant Cell Ther (2021) 27:334.e1–11. doi: 10.1016/j.jtct.2021.01.023 33836881

[27] Ringden O Labopin M Ciceri F Velardi A Bacigalupo A Arcese W. Is There a Stronger Graft-Versus-Leukemia Effect Using HLA-Haploidentical Donors Compared With HLA-Identical Siblings? Leukemia (2016) 30:447–55. doi: 10.1038/leu.2015.232 26293645

[28] Powles RL Morgenstern GR Kay HE McElwain TJ Clink HM Dady PJ. Mismatched Family Donors for Bone-Marrow Transplantation as Treatment for Acute Leukaemia. Lancet (1983) 1:612–5. doi: 10.1016/S0140-6736(83)91793-2 6131300

[29] Aversa F Tabilio A Terenzi A Velardi A Falzetti F Giannoni C. Successful Engraftment of T-Cell-Depleted Haploidentical “Three-Loci” Incompatible Transplants in Leukemia Patients by Addition of Recombinant Human Granulocyte Colony-Stimulating Factor-Mobilized Peripheral Blood Progenitor Cells to Bone Marrow Inoculum. Blood (1994) 84:3948–55. doi: 10.1182/blood.V84.11.3948.bloodjournal84113948 7524753

[30] Pierini A Ruggeri L Carotti A Falzetti F Saldi S Terenzi A. Haploidentical Age-Adapted Myeloablative Transplant and Regulatory and Effector T Cells for Acute Myeloid Leukemia. Blood Adv (2021) 5:1199–208. doi: 10.1182/bloodadvances.2020003739 PMC7948281 33646302

[31] Lu DP Dong L Wu T Huang XJ Zhang MJ Han W. Conditioning Including Antithymocyte Globulin Followed by Unmanipulated HLA-Mismatched/Haploidentical Blood and Marrow Transplantation Can Achieve Comparable Outcomes With HLA-Identical Sibling Transplantation. Blood (2006) 107:3065–73. doi: 10.1182/blood-2005-05-2146 16380454

[32] Luznik L O’Donnell PV Symons HJ Chen AR Leffell MS Zahurak M. HLA-Haploidentical Bone Marrow Transplantation for Hematologic Malignancies Using Nonmyeloablative Conditioning and High-Dose, Posttransplantation Cyclophosphamide. Biol Blood Marrow Transplant (2008) 14:641–50. doi: 10.1016/j.bbmt.2008.03.005 PMC2633246 18489989

[33] Luznik L Engstrom LW Iannone R Fuchs EJ. Posttransplantation Cyclophosphamide Facilitates Engraftment of Major Histocompatibility Complex-Identical Allogeneic Marrow in Mice Conditioned With Low-Dose Total Body Irradiation. Biol Blood Marrow Transplant (2002) 8:131–8. doi: 10.1053/bbmt.2002.v8.pm11939602 11939602

[34] Kong Y Wang Y Zhang YY Shi MM Mo XD Sun YQ. Prophylactic Oral NAC Reduced Poor Hematopoietic Reconstitution by Improving Endothelial Cells After Haploidentical Transplantation. Blood Adv (2019) 3:1303–17. doi: 10.1182/bloodadvances.2018029454 PMC6482364 31015207

[35] Zhou Y Cao L Guo H Hong Y Wang M Wang K. Th2 Polarization in Target Organs Is Involved in the Alleviation of Pathological Damage Mediated by Transplanting Granulocyte Colony-Stimulating Factor-Primed Donor T Cells. Sci China Life Sci (2021) 64:1087–96. doi: 10.1007/s11427-020-1754-6 32880861

[36] Cieri N Greco R Crucitti L Morelli M Giglio F Levati G. Post-Transplantation Cyclophosphamide and Sirolimus After Haploidentical Hematopoietic Stem Cell Transplantation Using a Treosulfan-Based Myeloablative Conditioning and Peripheral Blood Stem Cells. Biol Blood Marrow Transplant (2015) 21:1506–14. doi: 10.1016/j.bbmt.2015.04.025 26001696

[37] Aversa F Pierini A Ruggeri L Martelli MF Velardi A. The Evolution of T Cell Depleted Haploidentical Transplantation. Front Immunol (2019) 10:2769. doi: 10.3389/fimmu.2019.02769 31827475 PMC6890606

[38] Chang YJ Zhao XY Huang XJ. Granulocyte Colony-Stimulating Factor-Primed Unmanipulated Haploidentical Blood and Marrow Transplantation. Front Immunol (2019) 10:2516. doi: 10.3389/fimmu.2019.02516 31749802 PMC6842971

[39] Chang YJ Huang XJ. Haploidentical SCT: The Mechanisms Underlying the Crossing of HLA Barriers. Bone Marrow Transplant (2014) 49:873–9. doi: 10.1038/bmt.2014.19 24566712

[40] Canaani J Savani BN Labopin M Huang XJ Ciceri F Arcese W. Donor Age Determines Outcome in Acute Leukemia Patients Over 40 Undergoing Haploidentical Hematopoietic Cell Transplantation. Am J Hematol (2018) 93:246–53. doi: 10.1002/ajh.24963 29114918

[41] Wang Y Chang YJ Xu LP Liu KY Liu DH Zhang XH. Who is the Best Donor for a Related HLA Haplotype-Mismatched Transplant? Blood (2014) 124:843–50. doi: 10.1182/blood-2014-03-563130 24916508

[42] Chang YJ Luznik L Fuchs EJ Huang XJ. How do We Choose the Best Donor for T-Cell-Replete, HLA-Haploidentical Transplantation? J Hematol Oncol (2016) 9:35. doi: 10.1186/s13045-016-0265-2 27071449 PMC4830035

[43] Santoro N Labopin M Ciceri F Van Lint MT Nasso D Blaise D. Impact of Conditioning Intensity on Outcomes of Haploidentical Stem Cell Transplantation for Patients With Acute Myeloid Leukemia 45 Years of Age and Over. Cancer (2019) 125:1499–506. doi: 10.1002/cncr.31941 30620383

[44] Sun YQ Han TT Wang Y Yan CH Wang FR Wang ZD. Haploidentical Stem Cell Transplantation With a Novel Conditioning Regimen in Older Patients: A Prospective Single-Arm Phase 2 Study. Front Oncol (2021) 11:639502. doi: 10.3389/fonc.2021.639502 33718234 PMC7952870

[45] Dholaria B Labopin M Angelucci E Ciceri F Diez-Martin JL Bruno B. Impact of Total Body Irradiation- vs Chemotherapy-Based Myeloablative Conditioning on Outcomes of Haploidentical Hematopoietic Cell Transplantation for Acute Myelogenous Leukemia. Am J Hematol (2020). doi: 10.1002/ajh.25934 32656791

[46] How J Slade M Vu K DiPersio JF Westervelt P Uy GL. T Cell-Replete Peripheral Blood Haploidentical Hematopoietic Cell Transplantation With Post-Transplantation Cyclophosphamide Results in Outcomes Similar to Transplantation From Traditionally Matched Donors in Active Disease Acute Myeloid Leukemia. Biol Blood Marrow Transplant (2017) 23:648–53. doi: 10.1016/j.bbmt.2017.01.068 PMC5514180 28087457

[47] Arcuri LJ Hamerschlak N Rocha V Bonfim C Kerbauy MN. Outcomes After Haploidentical Cell Transplantation With Posttransplant Cyclophosphamide: A Systematic Review and Meta-Analysis Comparing Myeloablative With Reduced-Intensity Conditioning Regimen and Bone Marrow With Peripheral Blood Stem-Cell Graft: PTCy: PBSC or BM and MAC or RIC for Haplo-HCT With PTCy. Transplant Cell Ther (2021) 27:782.e1–782.e7. doi: 10.1016/j.jtct.2021.06.011 34146733

[48] Chang YJ Zhao XY Xu LP Zhang XH Wang Y Han W. Donor-Specific Anti-Human Leukocyte Antigen Antibodies Were Associated With Primary Graft Failure After Unmanipulated Haploidentical Blood and Marrow Transplantation: A Prospective Study With Randomly Assigned Training and Validation Sets. J Hematol Oncol (2015) 8:84. doi: 10.1186/s13045-015-0182-9 26156584 PMC4496923

[49] Chang YJ Xu LP Wang Y Zhang XH Chen H Chen YH. Rituximab for Desensitization During HLA-Mismatched Stem Cell Transplantation in Patients With a Positive Donor-Specific Anti-HLA Antibody. Bone Marrow Transplant (2020) 55:1326–36. doi: 10.1038/s41409-020-0928-z 32385341

[50] Al-Homsi AS Cole K Muilenburg M Goodyke A Abidi M Duffner U. Calcineurin and mTOR Inhibitor-Free Post-Transplantation Cyclophosphamide and Bortezomib Combination for Graft-Versus-Host Disease Prevention After Peripheral Blood Allogeneic Hematopoietic Stem Cell Transplantation: A Phase I/II Study. Biol Blood Marrow Transplant (2017) 23:1651–7. doi: 10.1016/j.bbmt.2017.05.024 28549771

[51] Chang YJ Xu LP Wang Y Zhang XH Chen d Chen YH. Controlled, Randomized, Open-Label Trial of Risk-Stratified Corticosteroid Prevention of Acute Graft-Versus-Host Disease After Haploidentical Transplantation. J Clin Oncol (2016) 34:1855–63. doi: 10.1200/JCO.2015.63.8817 27091717

[52] Gao L Zhang Y Hu B Liu J Kong P Lou S. Phase II Multicenter, Randomized, Double-Blind Controlled Study of Efficacy and Safety of Umbilical Cord-Derived Mesenchymal Stromal Cells in the Prophylaxis of Chronic Graft-Versus-Host Disease After HLA-Haploidentical Stem-Cell Transplantation. J Clin Oncol (2016) 34:2843–50. doi: 10.1200/JCO.2015.65.3642 27400949

[53] Yan CH Liu DH Liu KY Xu LP Liu YR Chen H. Risk Stratification-Directed Donor Lymphocyte Infusion Could Reduce Relapse of Standard-Risk Acute Leukemia Patients After Allogeneic Hematopoietic Stem Cell Transplantation. Blood (2012) 119:3256–62. doi: 10.1182/blood-2011-09-380386 22337715

[54] Chang YJ Zhao XY Huang XJ. Immune Reconstitution After Haploidentical Hematopoietic Stem Cell Transplantation. Biol Blood Marrow Transplant (2014) 20:440–9. doi: 10.1016/j.bbmt.2013.11.028 24315844

[55] Zhao XY Pei XY Chang YJ Yu XX Xu LP Wang Y. First-Line Therapy With Donor-Derived Human Cytomegalovirus (HCMV)-Specific T Cells Reduces Persistent HCMV Infection by Promoting Antiviral Immunity After Allogenic Stem Cell Transplantation. Clin Infect Dis (2020) 70:1429–37. doi: 10.1093/cid/ciz368 31067570

[56] Zhao XY Yu XX Xu ZL Cao XH Huo MR Zhao XS. Donor and Host Coexpressing KIR Ligands Promote NK Education After Allogeneic Hematopoietic Stem Cell Transplantation. Blood Adv (2019) 3:4312–25. doi: 10.1182/bloodadvances.2019000242 PMC6929384 31869417

[57] Esquirol A Pascual MJ Kwon M Perez A Parody R Ferra C. Severe Infections and Infection-Related Mortality in a Large Series of Haploidentical Hematopoietic Stem Cell Transplantation With Post-Transplant Cyclophosphamide. Bone Marrow Transplant (2021). doi: 10.1038/s41409-021-01328-4 PMC8165955 34059802

[58] Goldsmith SR Abid MB Auletta JJ Bashey A Beitinjaneh A Castillo P. Posttransplant Cyclophosphamide Is Associated With Increased Cytomegalovirus Infection: A CIBMTR Analysis. Blood (2021) 137:3291–305. doi: 10.1182/blood.2020009362 PMC8351903 33657221

[59] Lv M Wang Y Chang YJ Zhang XH Xu LP Jiang Q. Myeloablative Haploidentical Transplantation Is Superior to Chemotherapy for Patients With Intermediate-Risk Acute Myelogenous Leukemia in First Complete Remission. Clin Cancer Res (2019) 25:1737–48. doi: 10.1158/1078-0432.CCR-18-1637 30478089

[60] Xue YJ Cheng YF Lu AD Wang Y Zuo YX Yan CH. Efficacy of Haploidentical Hematopoietic Stem Cell Transplantation Compared With Chemotherapy as Postremission Treatment of Children With Intermediate-Risk Acute Myeloid Leukemia in First Complete Remission. Clin Lymphoma Myeloma Leuk (2021) 21:e126–e36. doi: 10.1016/j.clml.2020.09.004 33060049

[61] Hu GH Cheng YF Lu AD Wang Y Zuo YX Yan CH. Allogeneic Hematopoietic Stem Cell Transplantation can Improve the Prognosis of High-Risk Pediatric T(8;21) Acute Myeloid Leukemia in First Remission Based on MRD-Guided Treatment. BMC Cancer (2020) 20:553. doi: 10.1186/s12885-020-07043-5 32539815 PMC7294617

[62] Duan W Liu X Jia J Wang J Gong L Jiang Q. The Loss or Absence of Minimal Residual Disease of <0.1% at Any Time After Two Cycles of Consolidation Chemotherapy in CBFB-MYH11-Positive Acute Myeloid Leukaemia Indicates Poor Prognosis. Br J Haematol (2021) 192:265–71. doi: 10.1111/bjh.16745 32588434

[63] Yalniz FF Saliba RM Greenbaum U Ramdial J Popat U Oran B. Outcomes of Second Allogeneic Hematopoietic Cell Transplantation for Patients With Acute Myeloid Leukemia. Transplant Cell Ther (2021) 27:689–95. doi: 10.1016/j.jtct.2021.05.007 PMC8316329 34023569

[64] Battipaglia G Boumendil A Labopin M Ciceri F Tischer J Stelljes M. Unmanipulated Haploidentical Versus HLA-Matched Sibling Allogeneic Hematopoietic Stem Cell Transplantation in Relapsed/Refractory Acute Myeloid Leukemia: A Retrospective Study on Behalf of the ALWP of the EBMT. Bone Marrow Transplant (2019) 54:1499–510. doi: 10.1038/s41409-019-0459-7 30718798

[65] Fuchs EJ O’Donnell PV Eapen M Logan B Antin JH Dawson P. Double Unrelated Umbilical Cord Blood vs HLA-Haploidentical Bone Marrow Transplantation: The BMT CTN 1101 Trial. Blood (2021) 137:420–8. doi: 10.1182/blood.2020007535 PMC7819761 33475736

[66] Mehta RS Holtan SG Wang T Hemmer MT Spellman SR Arora M. Composite GRFS and CRFS Outcomes After Adult Alternative Donor HCT. J Clin Oncol (2020) 38:2062–76. doi: 10.1200/JCO.19.00396 PMC7302955 32364845

[67] Bashey A Zhang X Sizemore CA Manion K Brown S Holland HK. T-Cell-Replete HLA-Haploidentical Hematopoietic Transplantation for Hematologic Malignancies Using Post-Transplantation Cyclophosphamide Results in Outcomes Equivalent to Those of Contemporaneous HLA-Matched Related and Unrelated Donor Transplantation. J Clin Oncol (2013) 31:1310–6. doi: 10.1200/JCO.2012.44.3523 23423745

[68] Ruggeri A Labopin M Sanz G Piemontese S Arcese W Bacigalupo A. Comparison of Outcomes After Unrelated Cord Blood and Unmanipulated Haploidentical Stem Cell Transplantation in Adults With Acute Leukemia. Leukemia (2015) 29:1891–900. doi: 10.1038/leu.2015.98 25882700

[69] Luo Y Xiao H Lai X Shi J Tan Y He J. T-Cell-Replete Haploidentical HSCT With Low-Dose Anti-T-Lymphocyte Globulin Compared With Matched Sibling HSCT and Unrelated HSCT. Blood (2014) 124:2735–43. doi: 10.1182/blood-2014-04-571570 PMC4208287 25214441

[70] Versluis J Labopin M Ruggeri A Socie G Wu D Volin L. Alternative Donors for Allogeneic Hematopoietic Stem Cell Transplantation in Poor-Risk AML in CR1. Blood Adv (2017) 1:477–85. doi: 10.1182/bloodadvances.2016002386 PMC5738980 29296964

[71] Wu R Ma L. Haploidentical Hematopoietic Stem Cell Transplantation Versus Umbilical Cord Blood Transplantation in Hematologic Malignancies: A Systematic Review and Meta-Analysis. Cell Transplant (2020) 29:963689720964771. doi: 10.1177/0963689720964771 33040595 PMC7784570

[72] Dholaria B Labopin M Sanz J Ruggeri A Cornelissen J Labussiere-Wallet H. Allogeneic Hematopoietic Cell Transplantation With Cord Blood Versus Mismatched Unrelated Donor With Post-Transplant Cyclophosphamide in Acute Myeloid Leukemia. J Hematol Oncol (2021) 14:76. doi: 10.1186/s13045-021-01086-2 33941226 PMC8094558

[73] Nagler A Kanate AS Labopin M Ciceri F Angelucci E Koc Y. Post-Transplant Cyclophosphamide Versus Anti-Thymocyte Globulin for Graft-Versus-Host Disease Prevention in Haploidentical Transplantation for Adult Acute Lymphoblastic Leukemia. Haematologica (2021) 106:1591–8. doi: 10.3324/haematol.2020.247296 PMC8168508 32354866

[74] Salvatore D Labopin M Ruggeri A Battipaglia G Ghavamzadeh A Ciceri F. Outcomes of Hematopoietic Stem Cell Transplantation From Unmanipulated Haploidentical Versus Matched Sibling Donor in Patients With Acute Myeloid Leukemia in First Complete Remission With Intermediate or High-Risk Cytogenetics: A Study From the Acute Leukemia Working Party of the European Society for Blood and Marrow Transplantation. Haematologica (2018) 103:1317–28. doi: 10.3324/haematol.2018.189258 PMC6068036 29748438

[75] Kharfan-Dabaja MA Labopin M Brissot E Kroger N Finke J Ciceri F. Second Allogeneic Haematopoietic Cell Transplantation Using HLA-Matched Unrelated Versus T-Cell Replete Haploidentical Donor and Survival in Relapsed Acute Myeloid Leukaemia. Br J Haematol (2021) 193:592–601. doi: 10.1111/bjh.17426 33838047

[76] Xu LP Wu DP Han MZ Huang H Liu QF Liu DH. A Review of Hematopoietic Cell Transplantation in China: Data and Trends During 2008-2016. Bone Marrow Transplant (2017) 52:1512–8. doi: 10.1038/bmt.2017.59 28436973

[77] Passweg JR Baldomero H Chabannon C Basak GW de la Camara R Corbacioglu S. Hematopoietic Cell Transplantation and Cellular Therapy Survey of the EBMT: Monitoring of Activities and Trends Over 30 Years. Bone Marrow Transplant (2021). doi: 10.1038/s41409-021-01227-8 PMC8263343 33623153

[78] US 2020 Summary Slides of CIBMTR. Available at: https://www.cibmtr.org/ReferenceCenter/SlidesReports/SummarySlides/Documents/US%202020%20Summary%20Slides%20-%20final%20-%20for%20web%20posting.pptx.

[79] Rashidi A Hamadani M Zhang MJ Wang HL Abdel-Azim H Aljurf M. Outcomes of Haploidentical vs Matched Sibling Transplantation for Acute Myeloid Leukemia in First Complete Remission. Blood Adv (2019) 3:1826–36. doi: 10.1182/bloodadvances.2019000050 PMC6595262 31201170

[80] Baron F Labopin M Ruggeri A Cornelissen JJ Meijer E Sengeloev H. Impact of Donor Type in Patients With AML Given Allogeneic Hematopoietic Cell Transplantation After Low-Dose TBI-Based Regimen. Clin Cancer Res (2018) 24:2794–803. doi: 10.1158/1078-0432.CCR-17-3622 29555662

[81] Xiao-Jun H Lan-Ping X Kai-Yan L Dai-Hong L Yu W Huan C. Partially Matched Related Donor Transplantation can Achieve Outcomes Comparable With Unrelated Donor Transplantation for Patients With Hematologic Malignancies. Clin Cancer Res (2009) 15:4777–83. doi: 10.1158/1078-0432.CCR-09-0691 19584148

[82] Huang J Huang F Fan Z Xu N Xuan L Liu H. Haploidentical Related Donor vs Matched Sibling Donor Allogeneic Hematopoietic Stem Cell Transplantation for Acute Myeloid Leukemia and Myelodysplastic Syndrome Aged Over 50 Years: A Single-Center Retrospective Study. Cancer Med (2020) 9:6244–55. doi: 10.1002/cam4.3290 PMC7476836 32686915

[83] Santoro N Labopin M Giannotti F Ehninger G Niederwieser D Brecht A. Unmanipulated Haploidentical in Comparison With Matched Unrelated Donor Stem Cell Transplantation in Patients 60 Years and Older With Acute Myeloid Leukemia: A Comparative Study on Behalf of the ALWP of the EBMT. J Hematol Oncol (2018) 11:55. doi: 10.1186/s13045-018-0598-0 29661208 PMC5902953

[84] Ruggeri A Labopin M Savani B Paviglianiti A Blaise D Volt F. Hematopoietic Stem Cell Transplantation With Unrelated Cord Blood or Haploidentical Donor Grafts in Adult Patients With Secondary Acute Myeloid Leukemia, a Comparative Study From Eurocord and the ALWP EBMT. Bone Marrow Transplant (2019) 54:1987–94. doi: 10.1038/s41409-019-0582-5 31150016

[85] Brissot E Labopin M Ehninger G Stelljes M Brecht A Ganser A. Haploidentical Versus Unrelated Allogeneic Stem Cell Transplantation for Relapsed/Refractory Acute Myeloid Leukemia: A Report on 1578 Patients From the Acute Leukemia Working Party of the EBMT. Haematologica (2019) 104:524–32. doi: 10.3324/haematol.2017.187450 PMC6395335 30361416

[86] Sanz J Galimard JE Labopin M Afanasyev B Angelucci E Ciceri F. Post-Transplant Cyclophosphamide After Matched Sibling, Unrelated and Haploidentical Donor Transplants in Patients With Acute Myeloid Leukemia: A Comparative Study of the ALWP EBMT. J Hematol Oncol (2020) 13:46. doi: 10.1186/s13045-020-00882-6 32375860 PMC7201995

[87] Cho BS Yahng SA Min GJ Park S Park SS Shin SH. Comparable Outcomes After Alternative and Matched Sibling Donor Hematopoietic Stem Cell Transplantation and the Role of Molecular Measurable Residual Disease for Acute Myeloid Leukemia in Elderly Patients. Transplant Cell Ther (2021) 27:774.e1–12. doi: 10.1016/j.jtct.2021.05.024 34082159

[88] Zheng FM Zhang X Li CF Cheng YF Gao L He YL. Haploidentical- Versus Identical-Sibling Transplant for High-Risk Pediatric AML: A Multi-Center Study. Cancer Commun (Lond) (2020) 40:93–104. doi: 10.1002/cac2.12014 32175698 PMC7144412

[89] Ma R Huang XJ Xu LP Liu KY Zhang XH Yan CH. Comparable Outcomes After Hematopoietic Stem Cell Transplantation From Mother Donors and Matched Unrelated Donors in Patients With Hematopoietic Malignancies. Biol Blood Marrow Transplant (2019) 25:1210–7. doi: 10.1016/j.bbmt.2019.01.030 30708190

[90] Ma YR Xu LP Zhang XH Liu KY Chang YJ Lv M. Allogeneic Hematopoietic Stem Cell Transplantation for Intermediate-Risk Acute Myeloid Leukemia in the First Remission: Outcomes Using Haploidentical Donors are Similar to Those Using Matched Siblings. Ann Hematol (2021) 100:555–62. doi: 10.1007/s00277-020-04359-x 33415424

[91] Lu Y Zhao YL Zhang JP Xiong M Cao XY Liu DY. Comparable Outcomes Among Unmanipulated Haploidentical, Matched Unrelated, and Matched Sibling Donors in BU-Based Myeloablative Hematopoietic Stem Cell Transplantation for Intermediate and Adverse Risk Acute Myeloid Leukemia in Complete Remission: A Single-Center Study. Ann Hematol (2021) 100:1579–91. doi: 10.1007/s00277-020-04355-1 33236196

[92] Yu S Huang F Wang Y Xu Y Yang T Fan Z. Haploidentical Transplantation Might Have Superior Graft-Versus-Leukemia Effect Than HLA-Matched Sibling Transplantation for High-Risk Acute Myeloid Leukemia in First Complete Remission: A Prospective Multicentre Cohort Study. Leukemia (2020) 34:1433–43. doi: 10.1038/s41375-019-0686-3 31831845

[93] Yang B Yu R Cai L Bin G Chen H Zhang H. Haploidentical Versus Matched Donor Stem Cell Transplantation for Patients With Hematological Malignancies: A Systemic Review and Meta-Analysis. Bone Marrow Transplant (2019) 54:99–122. doi: 10.1038/s41409-018-0239-9 29988061

[94] Chang YJ Zhao XY Huo MR Xu LP Liu DH Liu KY. Immune Reconstitution Following Unmanipulated HLA-Mismatched/Haploidentical Transplantation Compared With HLA-Identical Sibling Transplantation. J Clin Immunol (2012) 32:268–80. doi: 10.1007/s10875-011-9630-7 22173879

[95] Gooptu M Romee R St Martin A Arora M Al Malki M Antin JH. HLA-Haploidentical vs Matched Unrelated Donor Transplants With Posttransplant Cyclophosphamide-Based Prophylaxis. Blood (2021) 138:273–82. doi: 10.1182/blood.2021011281 PMC8310426 34292325

[96] Arcuri LJ Aguiar MTM Ribeiro AAF Pacheco AGF. Haploidentical Transplantation With Post-Transplant Cyclophosphamide Versus Unrelated Donor Hematopoietic Stem Cell Transplantation: A Systematic Review and Meta-Analysis. Biol Blood Marrow Transplant (2019) 25:2422–30. doi: 10.1016/j.bbmt.2019.07.028 31386903

[97] Gagelmann N Bacigalupo A Rambaldi A Hoelzer D Halter J Sanz J. Haploidentical Stem Cell Transplantation With Posttransplant Cyclophosphamide Therapy vs Other Donor Transplantations in Adults With Hematologic Cancers: A Systematic Review and Meta-Analysis. JAMA Oncol (2019) 5:1739–48. doi: 10.1001/jamaoncol.2019.3541 PMC6802371 31621796

[98] Champlin R. Is Unrelated Donor or Haploidentical Hematopoietic Transplantation Preferred for Patients With Acute Myeloid Leukemia in Remission? Haematologica (2020) 105:252–4. doi: 10.3324/haematol.2019.239624 PMC7012488 32005652

[99] Brunstein CG Fuchs EJ Carter SL Karanes C Costa LJ Wu J. Alternative Donor Transplantation After Reduced Intensity Conditioning: Results of Parallel Phase 2 Trials Using Partially HLA-Mismatched Related Bone Marrow or Unrelated Double Umbilical Cord Blood Grafts. Blood (2011) 118:282–8. doi: 10.1182/blood-2011-03-344853 PMC3138683 21527516

[100] Kanate AS Szabo A Raj RV Bower K Grulke R Shah N. Comparison of Graft Acquisition and Early Direct Charges of Haploidentical Related Donor Transplantation Versus Umbilical Cord Blood Transplantation. Biol Blood Marrow Transplant (2019) 25:1456–64. doi: 10.1016/j.bbmt.2019.03.013 30878605

[101] Ruggeri A Sun Y Labopin M Bacigalupo A Lorentino F Arcese W. Post-Transplant Cyclophosphamide Versus Anti-Thymocyte Globulin as Graft- Versus-Host Disease Prophylaxis in Haploidentical Transplant. Haematologica (2017) 102:401–10. doi: 10.3324/haematol.2016.151779 PMC5286948 27758821

[102] Tang F Xu Y Chen H Xu L Zhang X Wang Y. Comparison of the Clinical Outcomes of Hematologic Malignancies After Myeloablative Haploidentical Transplantation With G-CSF/ATG and Posttransplant Cyclophosphamide: Results From the Chinese Bone Marrow Transplantation Registry Group (CBMTRG). Sci China Life Sci (2020) 63:571–81. doi: 10.1007/s11427-019-9594-7 31420852

[103] Zhou JR Shi DY Wei R Wang Y Yan CH Zhang XH. Co-Reactivation of Cytomegalovirus and Epstein-Barr Virus Was Associated With Poor Prognosis After Allogeneic Stem Cell Transplantation. Front Immunol (2020) 11:620891. doi: 10.3389/fimmu.2020.620891 33664733 PMC7921792

[104] Mulroney CM Bilal Abid M Bashey A Chemaly RF Ciurea SO Chen M. Incidence and Impact of Community Respiratory Viral Infections in Post-Transplant Cyclophosphamide-Based Graft-Versus-Host Disease Prophylaxis and Haploidentical Stem Cell Transplantation. Br J Haematol (2021) 194:145–57. doi: 10.1111/bjh.17563 PMC8853845 34124796

[105] Chang YJ Huang XJ. Is Human Leukocyte Antigen-Matched Sibling Donor Transplant Always Better Than Haploidentical Allograft? Semin Hematol (2019) 56:201–8. doi: 10.1053/j.seminhematol.2018.07.005 31202431

[106] Liu J Zhao XS Liu YR Xu LP Zhang XH Chen H. Association of Persistent Minimal Residual Disease With Poor Outcomes of Patients With Acute Myeloid Leukemia Undergoing Allogeneic Hematopoietic Stem Cell Transplantation. Chin Med J (Engl) (2018) 131:2808–16. doi: 10.4103/0366-6999.246072 PMC6278188 30511683

[107] Chang YJ Zhao XS Wang Y Liu YR Xu LP Zhang XH. Effects of Pre- and Post-Transplantation Minimal Residual Disease on Outcomes in Pediatric Patients With Acute Myeloid Leukemia Receiving Human Leukocyte Antigen-Matched or Mismatched Related Donor Allografts. Am J Hematol (2017) 92:E659–E61. doi: 10.1002/ajh.24910 28929514

[108] Chang YJ Wang Y Liu YR Xu LP Zhang XH Chen H. Haploidentical Allograft is Superior to Matched Sibling Donor Allograft in Eradicating Pre-Transplantation Minimal Residual Disease of AML Patients as Determined by Multiparameter Flow Cytometry: A Retrospective and Prospective Analysis. J Hematol Oncol (2017) 10:134. doi: 10.1186/s13045-017-0502-3 28676064 PMC5496245

[109] Xiaosu Z Leqing C Yazhen Q Yu W Xiaohui Z Lanping X. Classifying AML Patients With Inv(16) Into High-Risk and Low-Risk Relapsed Patients Based on Peritransplantation Minimal Residual Disease Determined by CBFbeta/MYH11 Gene Expression. Ann Hematol (2019) 98:73–81. doi: 10.1007/s00277-018-3480-9 30159599

[110] Zhao X Wang Z Ruan G Liu Y Wang Y Zhang X. Impact of Pre-Transplantation Minimal Residual Disease Determined by Multiparameter Flow Cytometry on the Outcome of AML Patients With FLT3-ITD After Allogeneic Stem Cell Transplantation. Ann Hematol (2018) 97:967–75. doi: 10.1007/s00277-018-3265-1 29423758

[111] Chang YJ Wang Y Xu LP Zhang XH Chen H Chen YH. Haploidentical Donor is Preferred Over Matched Sibling Donor for Pre-Transplantation MRD Positive ALL: A Phase 3 Genetically Randomized Study. J Hematol Oncol (2020) 13:27. doi: 10.1186/s13045-020-00860-y 32228710 PMC7106867

[112] Nagler A Labopin M Houhou M Aljurf M Mousavi A Hamladji RM. Outcome of Haploidentical Versus Matched Sibling Donors in Hematopoietic Stem Cell Transplantation for Adult Patients With Acute Lymphoblastic Leukemia: A Study From the Acute Leukemia Working Party of the European Society for Blood and Marrow Transplantation. J Hematol Oncol (2021) 14:53. doi: 10.1186/s13045-021-01065-7 33794963 PMC8017786

[113] Srour SA Saliba RM Bittencourt MCB Perez JMR Kongtim P Alousi A. Haploidentical Transplantation for Acute Myeloid Leukemia Patients With Minimal/Measurable Residual Disease at Transplantation. Am J Hematol (2019) 94:1382–7. doi: 10.1002/ajh.25647 31595538

[114] Wang T Chen S Chen J Liu T Zhang T Qiu H. Allogeneic Hematopoietic Stem Cell Transplantation Improved Survival for Adult Core Binding Factor Acute Myelogenous Leukemia Patients With Intermediate- and Adverse-Risk Genetics in the 2017 European LeukemiaNet. Transplant Cell Ther (2021) 27:173 e1– e9. doi: 10.1016/j.jtct.2020.10.010 33830030

[115] Kekre N Antin JH. Hematopoietic Stem Cell Transplantation Donor Sources in the 21st Century: Choosing the Ideal Donor When a Perfect Match Does Not Exist. Blood (2014) 124:334–43. doi: 10.1182/blood-2014-02-514760 24914138

[116] Little AM Akbarzad-Yousefi A Anand A Diaz Burlinson N Dunn PPJ Evseeva I. BSHI Guideline: HLA Matching and Donor Selection for Haematopoietic Progenitor Cell Transplantation. Int J Immunogenet (2021) 48:75–109. doi: 10.1111/iji.12527 33565720

[117] DeZern AE Franklin C Tsai HL Imus PH Cooke KR Varadhan R. Relationship of Donor Age and Relationship to Outcomes of Haploidentical Transplantation With Posttransplant Cyclophosphamide. Blood Adv (2021) 5:1360–8. doi: 10.1182/bloodadvances.2020003922 PMC7948266 33661299

[118] Seo S Usui Y Matsuo K Atsuta Y Igarashi A Fukuda T. Impact of the Combination of Donor Age and HLA Disparity on the Outcomes of Unrelated Bone Marrow Transplantation. Bone Marrow Transplant (2021) 56:2410–22. doi: 10.1038/s41409-021-01289-8 33990702

[119] Shimoni A Labopin M Lorentino F Van Lint MT Koc Y Gulbas Z. Killer Cell Immunoglobulin-Like Receptor Ligand Mismatching and Outcome After Haploidentical Transplantation With Post-Transplant Cyclophosphamide. Leukemia (2019) 33:230–9. doi: 10.1038/s41375-018-0170-5 29907809

[120] Ruggeri L Vago L Eikema DJ de Wreede LC Ciceri F Diaz MA. Natural Killer Cell Alloreactivity in HLA-Haploidentical Hematopoietic Transplantation: A Study on Behalf of the CTIWP of the EBMT. Bone Marrow Transplant (2021). doi: 10.1038/s41409-021-01259-0 33767404

[121] Zhao XY Chang YJ Zhao XS Xu LP Zhang XH Liu KY. Recipient Expression of Ligands for Donor Inhibitory KIRs Enhances NK-Cell Function to Control Leukemic Relapse After Haploidentical Transplantation. Eur J Immunol (2015) 45:2396–408. doi: 10.1002/eji.201445057 25952732

[122] Ciurea SO Al Malki MM Kongtim P Fuchs EJ Luznik L Huang XJ. The European Society for Blood and Marrow Transplantation (EBMT) Consensus Recommendations for Donor Selection in Haploidentical Hematopoietic Cell Transplantation. Bone Marrow Transplant (2020) 55:12–24. doi: 10.1038/s41409-019-0499-z 30833742

[123] DiNardo CD Pratz K Pullarkat V Jonas BA Arellano M Becker PS. Venetoclax Combined With Decitabine or Azacitidine in Treatment-Naive, Elderly Patients With Acute Myeloid Leukemia. Blood (2019) 133:7–17. doi: 10.1182/blood-2018-08-868752 30361262 PMC6318429

[124] Wei AH Strickland SA Jr. Hou JZ Fiedler W Lin TL Walter RB. Venetoclax Combined With Low-Dose Cytarabine for Previously Untreated Patients With Acute Myeloid Leukemia: Results From a Phase Ib/II Study. J Clin Oncol (2019) 37:1277–84. doi: 10.1200/JCO.18.01600 PMC6524989 30892988

[125] DiNardo CD Lachowiez CA Takahashi K Loghavi S Xiao L Kadia T. Venetoclax Combined With FLAG-IDA Induction and Consolidation in Newly Diagnosed and Relapsed or Refractory Acute Myeloid Leukemia. J Clin Oncol (2021) 39:2768–78. doi: 10.1200/JCO.20.03736 PMC8407653 34043428

[126] Yao S Jianlin C Yarong L Botao L Qinghan W Hongliang F. Donor-Derived CD123-Targeted CAR T Cell Serves as a RIC Regimen for Haploidentical Transplantation in a Patient With FUS-ERG+ AML. Front Oncol (2019) 9:1358. doi: 10.3389/fonc.2019.01358 31850234 PMC6901822

[127] Michelozzi IM Kirtsios E Giustacchini A. Driving CAR T Stem Cell Targeting in Acute Myeloid Leukemia: The Roads to Success. Cancers (Basel) (2021) 13:2816. doi: 10.3390/cancers13112816 34198742 PMC8201025

[128] Cortes JE Khaled S Martinelli G Perl AE Ganguly S Russell N. Quizartinib Versus Salvage Chemotherapy in Relapsed or Refractory FLT3-ITD Acute Myeloid Leukaemia (QuANTUM-R): A Multicentre, Randomised, Controlled, Open-Label, Phase 3 Trial. Lancet Oncol (2019) 20:984–97. doi: 10.1016/S1470-2045(19)30150-0 31175001

[129] DiNardo CD Stein EM Pigneux A Altman JK Collins R Erba HP. Outcomes of Patients With IDH1-Mutant Relapsed or Refractory Acute Myeloid Leukemia Receiving Ivosidenib Who Proceeded to Hematopoietic Stem Cell Transplant. Leukemia (2021). doi: 10.1038/s41375-021-01229-x PMC8464620 33772143

[130] Karol SE Alexander TB Budhraja A Pounds SB Canavera K Wang L. Venetoclax in Combination With Cytarabine With or Without Idarubicin in Children With Relapsed or Refractory Acute Myeloid Leukaemia: A Phase 1, Dose-Escalation Study. Lancet Oncol (2020) 21:551–60. doi: 10.1016/S1470-2045(20)30060-7 PMC7153631 32171069

[131] Pulte ED Norsworthy KJ Wang Y Xu Q Qosa H Gudi R. FDA Approval Summary: Gilteritinib for Relapsed or Refractory Acute Myeloid Leukemia With a FLT3 Mutation. Clin Cancer Res (2021) 27:3515–21. doi: 10.1158/1078-0432.CCR-20-4271 PMC8506653 33632926

[132] Tenold ME Moskoff BN Benjamin DJ Hoeg RT Rosenberg AS Abedi M. Outcomes of Adults With Relapsed/Refractory Acute Myeloid Leukemia Treated With Venetoclax Plus Hypomethylating Agents at a Comprehensive Cancer Center. Front Oncol (2021) 11:649209. doi: 10.3389/fonc.2021.649209 33777810 PMC7991747

[133] Uy GL Aldoss I Foster MC Sayre PH Wieduwilt MJ Advani AS. Flotetuzumab as Salvage Immunotherapy for Refractory Acute Myeloid Leukemia. Blood (2021) 137:751–62. doi: 10.1182/blood.2020007732 PMC7885824 32929488

[134] Zhang H Wang P Li Z He Y Gan W Jiang H. Anti-CLL1 Chimeric Antigen Receptor T-Cell Therapy in Children With Relapsed/Refractory Acute Myeloid Leukemia. Clin Cancer Res (2021) 27:3549–55. doi: 10.1158/1078-0432.CCR-20-4543 33832948

[135] Laszlo GS Gudgeon CJ Harrington KH Dell'Aringa J Newhall KJ Means GD. Cellular Determinants for Preclinical Activity of a Novel CD33/CD3 Bispecific T-Cell Engager (BiTE) Antibody, AMG 330, Against Human AML. Blood (2014) 123:554–61. doi: 10.1182/blood-2013-09-527044 PMC3901068 24311721

[136] Valent P Bauer K Sadovnik I Smiljkovic D Ivanov D Herrmann H. Cell-Based and Antibody-Mediated Immunotherapies Directed Against Leukemic Stem Cells in Acute Myeloid Leukemia: Perspectives and Open Issues. Stem Cells Transl Med (2020) 9:1331–43. doi: 10.1002/sctm.20-0147 PMC7581453 32657052

[137] Khaldoyanidi S Nagorsen D Stein A Ossenkoppele G Subklewe M. Immune Biology of Acute Myeloid Leukemia: Implications for Immunotherapy. J Clin Oncol (2021) 39:419–32. doi: 10.1200/JCO.20.00475 PMC8078464 33434043

[138] Kharfan-Dabaja MA Labopin M Polge E Polge E Nishihori T Bazarbachi A Finke J. Association of Second Allogeneic Hematopoietic Cell Transplant vs Donor Lymphocyte Infusion With Overall Survival in Patients With Acute Myeloid Leukemia Relapse. JAMA Oncol (2018) 4:1245–53. doi: 10.1001/jamaoncol.2018.2091 PMC6143013 30003233

[139] Cui Q Qian C Xu N Kang L Dai H Cui W. CD38-Directed CAR-T Cell Therapy: A Novel Immunotherapy Strategy for Relapsed Acute Myeloid Leukemia After Allogeneic Hematopoietic Stem Cell Transplantation. J Hematol Oncol (2021) 14:82. doi: 10.1186/s13045-021-01092-4 34034795 PMC8152118

[140] Wang Y Chen H Chen J Han M Hu J Jiong H. The Consensus on the Monitoring, Treatment, and Prevention of Leukemia Relapse After Allogeneic Hematopoietic Stem Cell Transplantation in China. Cancer Lett (2018) 438:63–75. doi: 10.1016/j.canlet.2018.08.030 30217562

[141] Gao L Zhang Y Wang S Kong P Su Y Hu J. Effect of rhG-CSF Combined With Decitabine Prophylaxis on Relapse of Patients With High-Risk MRD-Negative AML After HSCT: An Open-Label, Multicenter, Randomized Controlled Trial. J Clin Oncol (2020) 38:4249–59. doi: 10.1200/JCO.19.03277 PMC7768335 33108244

[142] Burchert A Bug G Fritz LV Finke J Stelljes M Rollig C. Sorafenib Maintenance After Allogeneic Hematopoietic Stem Cell Transplantation for Acute Myeloid Leukemia With FLT3-Internal Tandem Duplication Mutation (SORMAIN). J Clin Oncol (2020) 38:2993–3002. doi: 10.1200/JCO.19.03345 32673171

[143] Mathew NR Baumgartner F Braun L O'Sullivan D Thomas S Waterhouse M. Sorafenib Promotes Graft-Versus-Leukemia Activity in Mice and Humans Through IL-15 Production in FLT3-ITD-Mutant Leukemia Cells. Nat Med (2018) 24:282–91. doi: 10.1038/nm.4484 PMC6029618 29431743

[144] Jaiswal SR Chakraborty S Lakhchaura R Shashi P Mehta A Soni M. Early and Sustained Expansion of Adaptive Natural Killer Cells Following Haploidentical Transplantation and CTLA4Ig-Primed Donor Lymphocyte Infusions Dissociate Graft-Versus-Leukemia and Graft-Versus-Host Effects. Transplant Cell Ther (2021) 27:144–51. doi: 10.1016/j.jtct.2020.10.005 33830023

[145] Kerbauy MN Ribeiro AAF Arcuri LJ Kerbauy LN da Silva CC Camargo LFA. Clinical Impact of Multiple DNA Virus Infections in Nondepleted Haploidentical and Unrelated Allogeneic Hematopoietic Stem Cell Transplantation. Transpl Infect Dis (2021) 23:e13626. doi: 10.1111/tid.13626 33900012

[146] Degli-Esposti MA Hill GR. Immune Control of Cytomegalovirus Reactivation in Stem Cell Transplantation. Blood (2021). doi: 10.1182/blood.2020010028 34166512

[147] Gottlieb DJ Clancy LE Withers B McGuire HM Luciani F Singh M. Prophylactic Antigen-Specific T-Cells Targeting Seven Viral and Fungal Pathogens After Allogeneic Haemopoietic Stem Cell Transplant. Clin Transl Immunol (2021) 10:e1249. doi: 10.1002/cti2.1249 PMC7960021 33747509

[148] Olson A Lin R Marin D Rafei H Bdaiwi MH Thall PF. Third-Party BK Virus-Specific Cytotoxic T Lymphocyte Therapy for Hemorrhagic Cystitis Following Allotransplantation. J Clin Oncol (2021) 39:2710–9. doi: 10.1200/JCO.20.02608 PMC10166368 33929874

[149] Prockop S Doubrovina E Suser S Heller G Barker J Dahi P. Off-The-Shelf EBV-Specific T Cell Immunotherapy for Rituximab-Refractory EBV-Associated Lymphoma Following Transplantation. J Clin Invest (2020) 130:733–47. doi: 10.1172/JCI121127 PMC6994129 31689242

[150] van Galen P Hovestadt V Wadsworth Ii MH Hughes TK Griffin GK Battaglia S. Single-Cell RNA-Seq Reveals AML Hierarchies Relevant to Disease Progression and Immunity. Cell (2019) 176:1265–81.e24. doi: 10.1016/j.cell.2019.01.031 30827681 PMC6515904

[151] Schmalbrock LK Dolnik A Cocciardi S Strang E Theis F Jahn N. Clonal Evolution of Acute Myeloid Leukemia With FLT3-ITD Mutation Under Treatment With Midostaurin. Blood (2021) 137:3093–104. doi: 10.1182/blood.2020007626 PMC8233666 33598693

[152] Wesely J Kotini AG Izzo F Luo H Yuan H Sun J. Acute Myeloid Leukemia iPSCs Reveal a Role for RUNX1 in the Maintenance of Human Leukemia Stem Cells. Cell Rep (2020) 31:107688. doi: 10.1016/j.celrep.2020.107688 32492433 PMC7786450

[153] Sachs K Sarver AL Noble-Orcutt KE LaRue RS Antony ML Chang D. Single-Cell Gene Expression Analyses Reveal Distinct Self-Renewing and Proliferating Subsets in the Leukemia Stem Cell Compartment in Acute Myeloid Leukemia. Cancer Res (2020) 80:458–70. doi: 10.1158/0008-5472.CAN-18-2932 PMC7002190 31784425

[154] Duy C Li M Teater M Meydan C Garrett-Bakelman FE Lee TC. Chemotherapy Induces Senescence-Like Resilient Cells Capable of Initiating AML Recurrence. Cancer Discov (2021) 11:1542–61. doi: 10.1158/2159-8290.CD-20-1375 PMC8178167 33500244

[155] Wu J Xiao Y Sun J Sun H Chen H Zhu Y. A Single-Cell Survey of Cellular Hierarchy in Acute Myeloid Leukemia. J Hematol Oncol (2020) 13:128. doi: 10.1186/s13045-020-00941-y 32977829 PMC7517826

[156] Stetson LC Balasubramanian D Ribeiro SP Stefan T Gupta K Xu X. Single Cell RNA Sequencing of AML Initiating Cells Reveals RNA-Based Evolution During Disease Progression. Leukemia (2021). doi: 10.1038/s41375-021-01338-7 PMC8807029 34244611

[157] Crinier A Dumas PY Escaliere B Piperoglou C Gil L Villacreces A. Single-Cell Profiling Reveals the Trajectories of Natural Killer Cell Differentiation in Bone Marrow and a Stress Signature Induced by Acute Myeloid Leukemia. Cell Mol Immunol (2021) 18:1290–304. doi: 10.1038/s41423-020-00574-8 PMC8093261 33239726

[158] Demaree B Delley CL Vasudevan HN Peretz CAC Ruff D Smith CC. Joint Profiling of DNA and Proteins in Single Cells to Dissect Genotype-Phenotype Associations in Leukemia. Nat Commun (2021) 12:1583. doi: 10.1038/s41467-021-21810-3 33707421 PMC7952600

[159] Miles LA Bowman RL Merlinsky TR Csete IS Ooi AT Durruthy-Durruthy R. Single-Cell Mutation Analysis of Clonal Evolution in Myeloid Malignancies. Nature (2020) 587:477–82. doi: 10.1038/s41586-020-2864-x PMC7677169 33116311

[160] Morita K Wang F Jahn K Hu T Tanaka T Sasaki Y. Clonal Evolution of Acute Myeloid Leukemia Revealed by High-Throughput Single-Cell Genomics. Nat Commun (2020) 11:5327. doi: 10.1038/s41467-020-19119-8 33087716 PMC7577981

[161] Bazarbachi A Bug G Baron F Brissot E Ciceri F Dalle IA. Clinical Practice Recommendation on Hematopoietic Stem Cell Transplantation for Acute Myeloid Leukemia Patients With FLT3-Internal Tandem Duplication: A Position Statement From the Acute Leukemia Working Party of the European Society for Blood and Marrow Transplantation. Haematologica (2020) 105:1507–16. doi: 10.3324/haematol.2019.243410 PMC7271578 32241850

[162] Zaghi E Calvi M Puccio S Spata G Terzoli S Peano C. Single-Cell Profiling Identifies Impaired Adaptive NK Cells Expanded After HCMV Reactivation in Haploidentical HSCT. JCI Insight (2021) 6:e146973. doi: 10.1172/jci.insight.146973 PMC8262468 34003794

[163] Zhao Y Gao F Wu Y Shi J Luo Y Tan Y. Decreased iKIR-HLA C Pair Confers Worse Clinical Outcomes for Patients With Myeloid Disease Receiving Antithymocyte Globulin-Based Haploidentical Hematopoietic Stem Cell Transplantation. Front Immunol (2020) 11:614488. doi: 10.3389/fimmu.2020.614488 33633734 PMC7901980

[164] Maffini E Labopin M Blaise D Ciceri F Gulbas Z Deconinck E. CD34+ Cell Dose Effects on Clinical Outcomes After T-Cell Replete Haploidentical Allogeneic Hematopoietic Stem Cell Transplantation for Acute Myeloid Leukemia Using Peripheral Blood Stem Cells. A Study From the Acute Leukemia Working Party of the European Society for Blood and Marrow Transplantation (EBMT). Am J Hematol (2020) 95:892–9. doi: 10.1002/ajh.25826 32303111

[165] Klyuchnikov E Badbaran A Massoud R Fritsche-Friedland U Janson D Ayuk F. Enhanced Immune Reconstitution of Gammadelta T Cells After Allogeneic Stem Cell Transplantation Overcomes the Negative Impact of Pretransplantation Minimal Residual Disease-Positive Status in Patients With Acute Myelogenous Leukemia. Transplant Cell Ther (2021) 27(10):841–50. doi: 10.1016/j.jtct.2021.06.003 34118468

